# Nanomedicine‐Driven Modulation of Reductive Stress for Cancer Therapy

**DOI:** 10.1002/advs.202501968

**Published:** 2025-06-26

**Authors:** Yumin Mao, Xianping Liu, Xu Chu, Feixiang Chen, Ruicheng Shi, Zonghao Liu, Yelin Wu, Yanyan Liu, Wenbo Bu

**Affiliations:** ^1^ Department of Materials Science and State Key Laboratory of Molecular Engineering of Polymers Academy for Engineering and Technology Fudan University Shanghai 200433 P. R. China; ^2^ Institute of Hepatobiliary and Pancreatic Surgery Department of Hepatobiliary and Pancreatic Surgery Shanghai East Hospital School of Medicine Tongji University Shanghai 200120 P. R. China

**Keywords:** nanomedicine, oxidative stress, redox balance, reductive stress, tumor treatment

## Abstract

Redox balance is crucial for cellular function and adaptation to environmental changes, with its disruption playing a key role in the progression of various diseases, including cancer. While oxidative stress caused by excessive reactive oxygen species (ROS) has been widely studied and targeted in cancer therapies, such approaches face significant challenges within the tumor microenvironment. On the opposite end, reductive stress results from an overabundance of reducing equivalents, disrupting normal ROS‐dependent signaling pathways and leading to cellular dysfunction. Despite its importance in tumor biology, reductive stress has received less attention than oxidative stress. This group has deliberately driven tumors into a state of reductive stress, thereby exposing unique vulnerabilities and validating a novel therapeutic strategy. Here, the concept and mechanisms of reductive stress is reviewed, introduced methods for detecting it, and discussed its dual role in tumor progression and potential as a therapeutic target. Recent advances in nanomedicine, particularly in the design of functional nanomaterials, enabling precise modulation of cellular redox states are also highlighted. By selectively inducing reductive stress within tumors, nanomedicine offers a promising strategy to exploit tumor vulnerabilities, overcome drug resistance, and improve cancer therapy efficacy.

## Graphical abstract

This article introduces a novel therapeutic paradigm that deliberately induces reductive stress within tumor cells to exploit their unique biochemical vulnerabilities. It systematically reviews the underlying biological mechanisms, current detection and monitoring techniques, and emerging advanced nanotechnology platforms specifically designed to modulate intracellular redox balance, offering promising new avenues for targeted and precise cancer treatment.

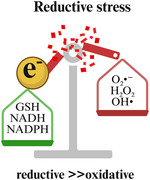

## 1 Introduction

Redox homeostasis is fundamental to cell function and survival, and its dysregulation is a hallmark of cancer progression.^[^ ^1^ ^]^ Tumor cells experience high levels of reactive oxygen species (ROS) due to their hypermetabolic state, yet excessive ROS can damage DNA, proteins, and lipids.^[^ ^2^ ^]^ Many current cancer therapies harness oxidative stress to trigger tumor cell death, for instance, by elevating ROS or by inhibiting antioxidants.^[^ ^3^ ^]^ However, the efficacy of solely ROS‐based strategies is limited by the tumor microenvironment; hypoxia and other factors enable cancer cells to resist oxidative damage and develop drug resistance. At the opposite end of the redox spectrum lies reductive stress, a state of redox imbalance caused by an overabundance of reducing equivalents (e.g. NADH, NADPH, GSH) and abnormally low ROS levels. Reductive stress has been implicated in various pathologies, such as neurodegeneration, cardiomyopathy, and cancer, but its precise role in tumors remains less understood, and until recently, research in this area was relatively underexplored due to limitations in both detection and modulation techniques.^[^ ^4^ ^]^ Traditionally, antioxidants and other reductants were presumed universally beneficial for cancer patients, aiming to neutralize ROS and mitigate therapy side effects. Counterintuitively, certain antioxidant supplements (e.g., high‐dose β‐carotene in smokers) have increased cancer incidence.^[^ ^5^ ^]^ These contradictory findings challenge the notion that more reducing power is always beneficial and hint that an overly reduced intracellular environment can be as deleterious as oxidative stress.

Indeed, beyond a threshold, an excess of reductants can disrupt ROS‐dependent signaling and organelle function, ultimately leading to cell damage or death. Reductive stress thus emerges as a double‑edged sword in cancer biology, capable of both promoting tumor survival and creating vulnerabilities in cancer cells. By tipping the redox balance in cancer cells further toward a reductive extreme, one can potentially exploit tumor‐specific vulnerabilities (such as dysregulated antioxidant systems and redox‐sensitive metabolic bottlenecks) to trigger cell death without harming normal tissues. Nanomedicine offers a cutting‐edge approach to achieve this selective redox modulation. Nanoscale drug delivery systems can accumulate in tumors, protect and concentrate redox‐active payloads, and release them in response to tumor‐specific triggers. Such precision targeting addresses the “5R” principles (Right species, Right place, Right time, Right level, Right target) proposed for redox interventions, which conventional systemic therapies struggle to fulfill.^[^ ^6^ ^]^ Building on these insights, our group was the first to pioneer and validate a reductive stress‐driven anticancer strategy. We used the photo‐generated electrons that were delivered into the endoplasmic reticulum (ER) of tumor cells, forcibly shifting the oxidative protein‐folding environment toward an excessively reductive state. This perturbation disrupts essential disulfide bonds and markedly increases intracellular levels of NADH and GSH, ultimately initiating a reductive stress‐induced cell death cascade.^[^ ^7^ ^]^

This review focuses on the role of reductive stress in cancer and how nanomedicine can be harnessed to modulate this stress for therapeutic benefit. We first outline the biological basis of reductive stress and its relevance to tumor physiology. We then discuss methods to detect and monitor reductive stress, a prerequisite for both understanding and targeting it. Next, we describe emerging nanomedicine strategies to induce or exploit reductive stress in cancer cells, highlighting representative examples and their mechanistic implications. Finally, we consider current limitations, potential pitfalls, and future directions in this nascent field, envisioning how nanomedicine‐driven redox modulation could advance precision cancer therapy.

## 2 Reductive Stress in Cancer

### 2.1 The Concept of Reductive Stress

**Figure 1:**
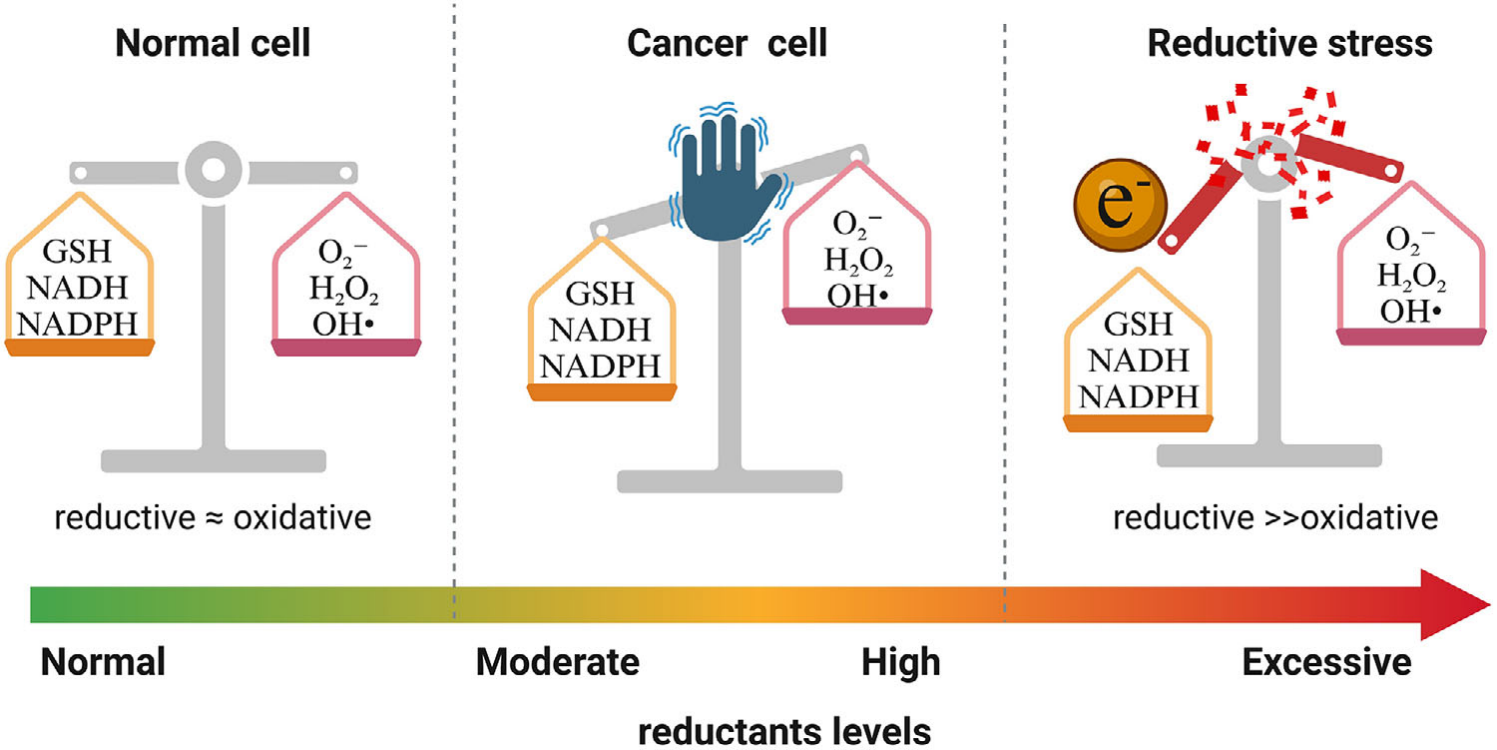
Schematic illustration of redox imbalance and reductive stress in cancer cells. In normal cells, the levels of antioxidants are balanced with ROS, ensuring proper cellular function. In cancer cells, the high metabolic rate leads to increased ROS production. To compensate, cancer cells upregulate reductive equivalents, resulting in an oscillating redox equilibrium. Excessive reductive stress in cancer cells can further disrupt redox balance, leading to cellular damage and dysfunction. Illustrations made using Biorender.com.

Reductive stress was first introduced in 1989 by Gores et al., who described metabolic disturbances occurring under hypoxic conditions or impaired mitochondrial respiration.^[^ ^8^ ^]^ In 2007, Benjamin et al. further refined this concept by demonstrating that excessive reductive levels in cardiomyocytes could lead to cellular damage, formally naming the phenomenon “reductive stress”.^[^ ^9^ ^]^ The term now refers to a state of redox imbalance marked by abnormally high levels of reducing equivalents and an overly reduced intracellular environment (**Figure** **1**). In essence, just as oxidative stress denotes an excess of ROS, reductive stress denotes an excess of antioxidants. Under physiological conditions, cells maintain a dynamic redox balance: organelles such as mitochondria continuously generate ROS, whereas antioxidant systems, including GSH and superoxide dismutase (SOD), promptly eliminate these reactive intermediates.^[^ ^10^ ^]^ This tightly regulated homeostasis ensures proper cell signaling and metabolism, thereby supporting normal cell function, proliferation, senescence, and orderly apoptosis. When this balance tips toward oxidative stress, elevated ROS levels damage proteins, lipids, and DNA, potentially triggering apoptosis or necrosis via pathways like p53 activation.^[^ ^11^ ^]^ Oxidative stress has been implicated in diverse diseases, including neurodegeneration and chronic inflammation, where it disrupts tissue integrity and function.^[^ ^12^ ^]^ Notably, in certain neurodegenerative contexts, a reductive shift can initially impair cellular function and eventually trigger secondary oxidative stress. Conversely, reductive stress represents the opposite end of the spectrum, an overly reduced intracellular environment. While a modest increase in reducing power can protect cells from oxidative damage, excessive reductive stress impairs signaling and protein folding. Thus, both oxidative and reductive stress reflect pathological deviations from redox homeostasis, and both can redirect cell fate depending on their severity and context.

**Figure 2:**
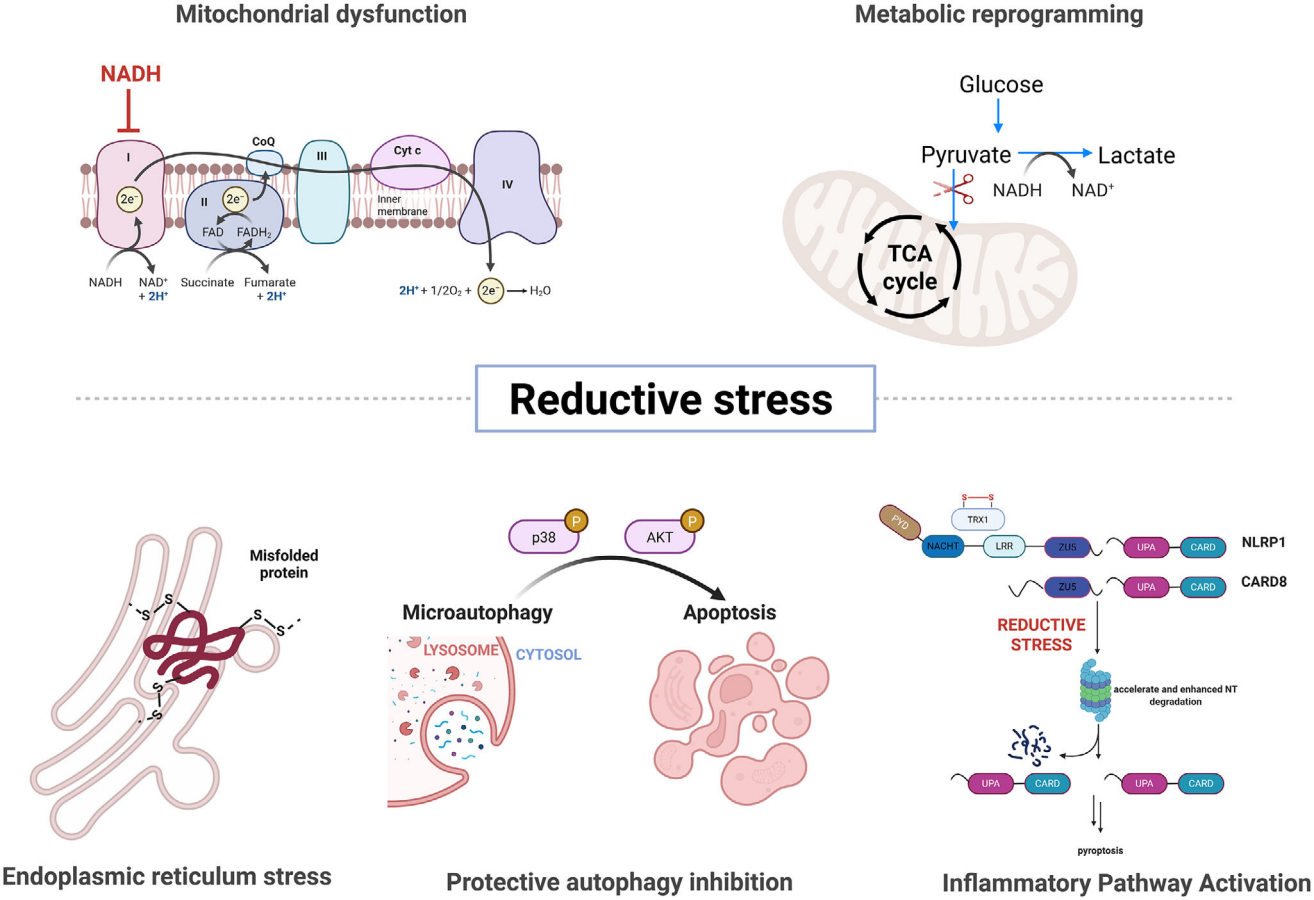
Mechanisms of reductive stress. The build‐up of NADH, NADPH, and GSH forces tumor cells into a hyper‑reduced state that stalls mitochondrial respiration, rewires central metabolism, disrupts ER protein folding, blocks protective autophagy, and over‑activates inflammasomes, ultimately driving metabolic collapse and cell death. Illustrations made using Biorender.com.

Notably, cancer cells often exist near a critical tipping point between oxidative and reductive extremes.^[^ ^11^, ^13^ ^]^ Due to their rapid proliferation and consequent high levels of ROS generation, tumor cells simultaneously enhance their antioxidant defenses,^[^ ^11^, ^14^ ^]^ resulting in a finely balanced redox state where only a moderately reduced intracellular environment effectively supports their growth and survival. Pushing this equilibrium further toward reduction collapses essential metabolic and signaling pathways, triggering lethal proteotoxicity and energy failure. Exploiting this vulnerability underpins the therapeutic rationale for reductive stress therapy.^[^ ^15^ ^]^ Unlike oxidative stress treatments such as radiotherapy, photodynamic therapy, or many chemotherapeutics,^[^ ^12a^ ^]^ which lose efficacy in hypoxic tumors^[^ ^16^ ^]^ and generate off‐target ROS that harm healthy tissues,^[^ ^17^ ^]^ treatments that induce reductive stress operate independently of oxygen levels and selectively target cells situated close to the reductive threshold. In other words, a mildly reduced state may promote tumor cell survival and proliferation (by buffering ROS and supporting anabolic metabolism), but an overly reduced state will interfere with essential ROS‐dependent signals and metabolic processes, creating vulnerabilities (**Figure** **2**).

#### 2.1.1 Mitochondrial Dysfunction

Excessive accumulation of reducing equivalents (e.g., NADH) disrupts mitochondrial respiration. When NADH builds up beyond the electron transport chain (ETC)'s capacity, it inhibits essential respiratory enzymes, causing electrons to leak prematurely and directly reduce oxygen, leading to bursts of ROS.^[^ ^18^ ^]^ This paradoxical generation of ROS, occurring amid NADH overload, can damage mitochondrial components.^[^ ^19^ ^]^ Additionally, a high NADH/NAD^+^ ratio exerts feedback inhibition on the tricarboxylic acid (TCA) cycle and pyruvate dehydrogenase, impairing ATP synthesis and triggering an energy crisis.^[^ ^18^ ^]^ Consequently, excessively reductive conditions drive mitochondrial dysfunction, depriving cells of critical energy and occasionally provoking sudden ROS surges that ultimately induce cell death.

#### 2.1.2 Metabolic Reprogramming

Accumulation of NADH stabilizes hypoxia‐inducible factor 1α (HIF‐1α) even under normoxic conditions,^[^ ^20^ ^]^ a phenomenon known as “pseudohypoxia.” This stabilization shifts cellular metabolism toward glycolysis (the Warburg effect),^[^ ^21^ ^]^ reducing reliance on oxidative phosphorylation. Although enhanced glycolysis partially regenerates NAD^+^ by converting pyruvate to lactate, it simultaneously worsens NADH/NAD^+^ imbalances, particularly in hypoxic regions of tumors.^[^ ^22^ ^]^ In the cytosol, an excessively reduced NADH pool can falsely signal nutrient abundance, promoting reductive biosynthesis pathways. For instance, elevated NADH (along with NADPH) can stimulate lipogenesis and nucleotide synthesis, initially supporting rapid cellular proliferation.^[^ ^23^ ^]^ However, once a critical threshold is surpassed, these metabolic adaptations become detrimental: vital oxidative reactions are impaired, leading to abnormal accumulation of metabolic intermediates.^[^ ^24^ ^]^ Ultimately, reductive stress traps cells in a metabolic bottleneck, limiting their metabolic flexibility and forcing reliance on glycolysis, which yields ATP inefficiently.^[^ ^25^ ^]^

#### 2.1.3 Protein Misfolding

The ER requires an oxidizing environment to ensure proper disulfide bond formation in newly synthesized proteins.^[^ ^26^ ^]^ An excessively reduced cytosolic environment disrupts this delicate redox balance, leading to improper formation or complete failure of disulfide bonds.^[^ ^27^ ^]^ Consequently, misfolded or unfolded proteins accumulate in the ER, activating the unfolded protein response (UPR) and causing ER stress.^[^ ^28^ ^]^ Persistent reductive stress further promotes abnormal protein aggregation. For example, in mammalian cells, prolonged reductive stress can induce aberrant aggregation of nuclear lamina proteins.^[^ ^29^ ^]^ This collapse in protein homeostasis not only impairs normal cellular functions but may also trigger cell death pathways if protein aggregates overwhelm the cell's chaperone systems and proteasomal degradation capacity.^[^ ^30^ ^]^

#### 2.1.4 Protective Autophagy Inhibition

A persistently reductive environment can inhibit essential cellular recovery mechanisms. Protective autophagy, a process crucial for cell survival^[^ ^31^ ^]^ by removing damaged organelles and proteins,^[^ ^32^ ^]^ is often suppressed under reductive stress. Research has demonstrated decreased expression of autophagy markers (e.g., Beclin1 and LC3B) alongside the increased accumulation of p62/SQSTM1 in cells undergoing chronic reductive conditions. Excess p62 further exacerbates cellular stress by stabilizing NF‐E2‐related factor 2 (NRF2), a transcription factor that elevates antioxidant levels, thereby perpetuating a feedback loop of sustained reductive signaling. Ultimately, cells experiencing prolonged reductive stress lose a vital mechanism for self‐clearance, becoming incapable of effectively removing damaged cellular components and consequently becoming more vulnerable to apoptosis when faced with additional stressors.^[^ ^32^ ^]^

#### 2.1.5 Inflammatory Pathway Activation

An over‐reduced intracellular environment can aberrantly activate certain inflammatory pathways. Thioredoxin (Trx), a major redox regulator, normally binds to and represses components of inflammasomes (multi‐protein complexes that trigger inflammatory cell death).^[^ ^33^ ^]^ Under reductive stress, Trx remains fully reduced and is unable to bind its targets, leading to uninhibited inflammasome assembly.^[^ ^34^ ^]^ This can provoke excessive maturation of IL‐1β/IL‐18 and lead to pyroptosis, a form of inflammatory cell death. Thus, reductive stress can paradoxically trigger inflammation rather than dampen it (as one might expect from lower ROS levels) by disabling redox‐sensitive inhibitors of inflammatory pathways.^[^ ^35^ ^]^ It can unleash it by disabling redox‐sensitive inhibitors of inflammation. This pro‐inflammatory cell death further contributes to tissue damage.^[^ ^34^ ^]^

Collectively, mitochondrial failure, metabolic collapse, protein misfolding, blocked autophagy, and hyperactive inflammasomes show how deep reductive stress nudges cells toward death. Tumors exist in a precarious balance: they elevate antioxidants to manage ROS, yet fine‐tuning makes them susceptible to collapse if the reducing load spikes. Push them just past their limit and a burst of electron transport chain (ETC)‐derived ROS or a wave of misfolded proteins can trigger selective cell death, which explains the appeal of therapeutically inducing “toxic reductive stress.” The key is to overload the tumor's reductive machinery rather than merely bolster it. The following sections will discuss the sources of cellular reducing power and why certain tumors are especially sensitive to reductive stress.

### 2.2 Reducing Equivalents in Cells

**Figure 3:**
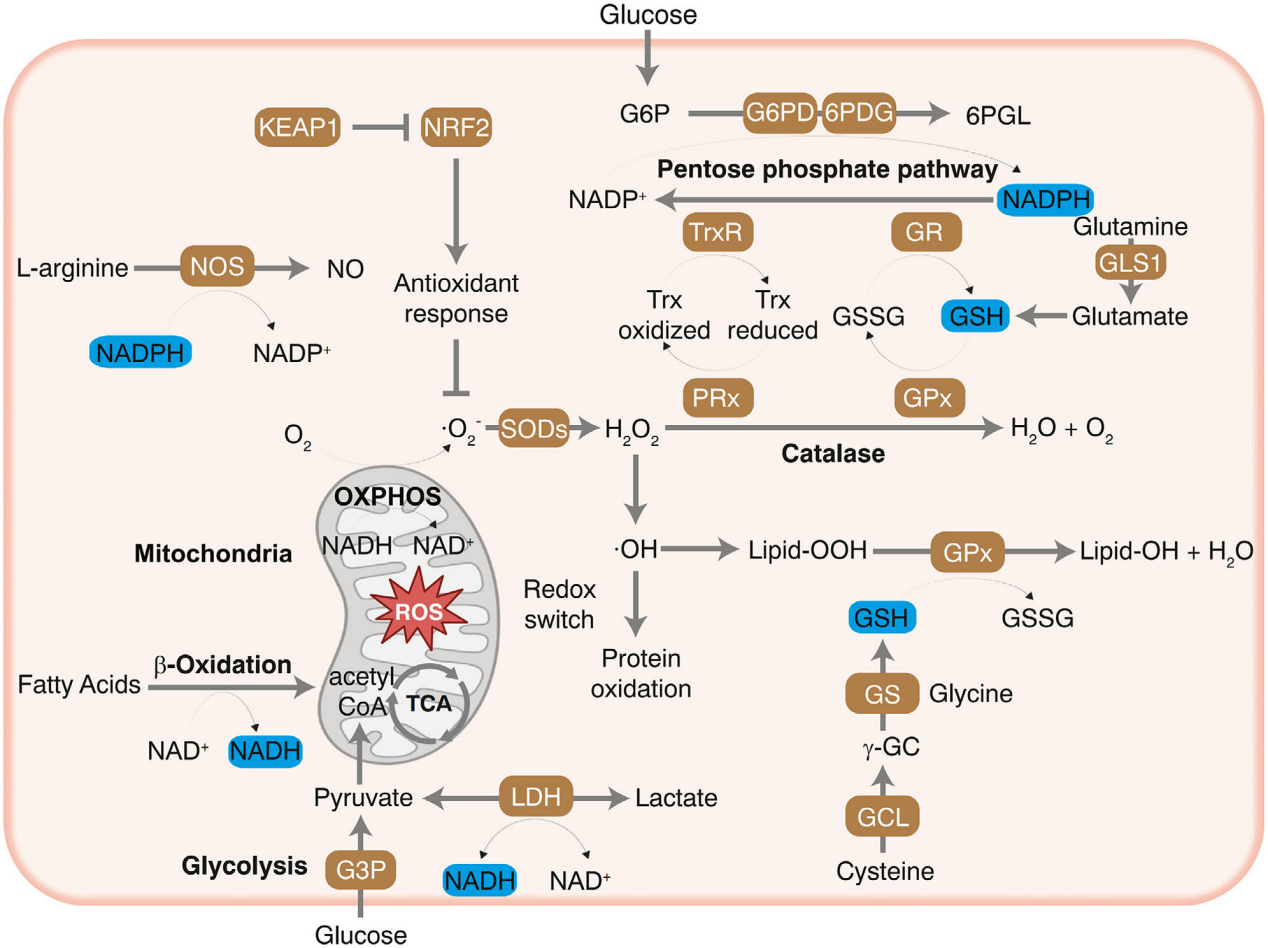
NAD(P)H‐ and GSH‐centered metabolic circuitry that preserves redox balance in cells. To maintain redox homeostasis, both nucleotide cofactors and antioxidants have coordinated effects, which are regulated by antioxidant response genes. NAD(P)H and GSH serve as the reducing power within cells to power antioxidant enzymes and quench certain ROS, respectively. ROS production from mitochondrial oxidative phosphorylation (OXPHOS) requires NADH to serve as the electron donor. Reproduced with permission.^[^ ^37^ ^]^ Copyright 2024, Elsevier.

To comprehend reductive stress, it is crucial to identify the primary reducing equivalents responsible for maintaining intracellular redox homeostasis. Cells contain several essential molecules that function as electron donors (reducing power) in metabolic processes and antioxidant defense systems. The primary intracellular reductants include the pyridine nucleotides NADH and NADPH, and small‐molecule thiols like GSH along with protein thiols exemplified by the Trx system.^[^ ^36^ ^]^ These species play indispensable roles in energy metabolism, biosynthesis, and redox signaling (**Figure** **3**).^[^ ^37^ ^]^ Dysregulation or overabundance of these reducing equivalents in tumors can tip cells into reductive stress.

#### 2.2.1 NADH (Nicotinamide Adenine Dinucleotide, Reduced Form)

NADH is a central electron carrier in cellular metabolism, primarily produced by catabolic processes.^[^ ^38^ ^]^ In normal cells, glycolysis, the TCA cycle, and β‐oxidation generate NADH, which mitochondria quickly convert back to NAD^+^ through oxidative phosphorylation, keeping the cytosolic NAD^+^/NADH ratio high (≈700:1).^[^ ^39^ ^]^ Cancer cells break this balance. The Warburg effect and tumor hypoxia accelerate glycolytic NADH production while limiting mitochondrial re‐oxidation, causing the NADH/NAD⁺ ratio to rise.^[^ ^21^ ^]^ As discussed above, excess NADH disrupts mitochondrial function and promotes metabolic reprogramming.^[^ ^18^, ^21^ ^]^ Thus, NADH underwrites growth but, when in excess, drives reductive stress.

#### 2.2.2 NADPH (Nicotinamide Adenine Dinucleotide Phosphate, Reduced Form)

NADPH is a crucial cellular redox cofactor structurally similar to NADH but containing a phosphate group that directs it to specific roles.^[^ ^40^ ^]^ It is primarily generated through the pentose phosphate pathway (PPP) via glucose‐6‐phosphate dehydrogenase and 6‐phosphogluconate dehydrogenase, as well as by other enzymes such as malic enzyme and cytosolic isocitrate dehydrogenase (IDH). Cells maintain a high NADPH/NADP^+^ ratio (> 100:1) ensuring NADPH availability for biosynthesis and antioxidant defense.^[^ ^41^ ^]^ NADPH supplies the reducing power necessary for fatty acid, cholesterol, and nucleotide synthesis and is essential for antioxidant systems, including regeneration of reduced GSH from GSSG by GSH reductase and maintaining Trx in its reduced form through Trx reductase.^[^ ^39^, ^41^ ^]^ Thus, NADPH facilitates peroxide detoxification and maintains a reduced intracellular environment. In cancer, NADPH supports the high anabolic demands and helps neutralize ROS generated during rapid proliferation.^[^ ^42^ ^]^ Tumor cells frequently upregulate NADPH‐producing pathways; for example, NRF2 activation increases PPP and malic enzyme activity,^[^ ^43^ ^]^ while oncogenes such as mutant IDH1 consume NADPH to produce 2‐HG,^[^ ^44^ ^]^ and MYC and KRAS boost NADPH production through metabolic reprogramming.^[^ ^45^ ^]^ Adequate NADPH levels allow cancer cells to counter oxidative stress and resist ROS‐inducing therapies. However, excessive NADPH accumulation can lead to reductive stress if unbalanced by ROS. This imbalance, often seen in NRF2‐hyperactive tumors, correlates with elevated NADPH and GSH levels.^[^ ^24^ ^]^ In summary, NADPH generation and consumption are tightly regulated, and disruptions can shift cellular redox balance toward oxidative or reductive extremes, significantly affecting cancer cell survival.

#### 2.2.3 GSH (Glutathione)

GSH is the most abundant non‐protein thiol in cells and acts as a major antioxidant.^[^ ^39a^ ^]^ It is a tripeptide (γ‐glutamyl‐cysteinyl‐glycine) that cycles between a reduced thiol form GSH and an oxidized disulfide‐bonded dimer (GSSG).^[^ ^46^ ^]^ GSH can directly scavenge reactive oxygen/nitrogen species and is a cofactor for enzymes like GSH peroxidases and GSH S‐transferases, which detoxify peroxides and xenobiotics.^[^ ^47^ ^]^ Cancer cells often have elevated GSH levels to cope with their oxidative stress; many tumors upregulate GSH biosynthesis or import cystine (via SLC7A11) to fuel GSH production.^[^ ^48^ ^]^ High GSH helps tumor cells resist chemotherapy and radiation (which kill cancer cells by generating ROS) by quenching those ROS. However, if GSH and other thiols accumulate excessively, the cell's redox environment can become too reducing. An overabundance of GSH skews the GSH/GSSG balance strongly toward the reduced state, contributing to reductive stress. One consequence of a highly reduced thiol pool is the disruption of disulfide bond formation in proteins (as noted above).^[^ ^49^ ^]^ Another is thiol buffering of ROS to the point that signaling pathways that rely on ROS are blunted.^[^ ^50^ ^]^ GSH is synthesized by glutamate cysteine ligase (GCL) and GSH synthetase; these enzymes are regulated by NRF2 and other stress‐response pathways.^[^ ^51^ ^]^ Tumors with mutations that constitutively activate NRF2 (e.g. Kelch‐like ECH‐associated protein 1 (KEAP1) mutations) often show dramatically elevated GSH content.^[^ ^52^ ^]^ While these cells can survive otherwise lethal oxidative conditions, they become susceptible to redox imbalance under elevated reducing conditions.^[^ ^24^ ^]^ In summary, GSH is essential for cellular redox homeostasis and drug resistance in cancer, yet its overabundance is a hallmark of reductive stress that can create vulnerabilities.

#### 2.2.4 Trx System

Complementing the GSH system, the Trx system represents a crucial parallel antioxidant network primarily focused on the reduction of disulfide bonds within proteins.^[^ ^53^ ^]^ This system is centered around the Trx proteins, notably Trx1, located in the cytosol and nucleus, and Thioredoxin‐2 (Trx2) within the mitochondria.^[^ ^54^ ^]^ These are relatively small proteins characterized by a conserved active site dithiol motif (‐Cys‐Gly‐Pro‐Cys‐), which directly facilitates the reduction of oxidized cysteine residues (disulfides) in a wide array of target proteins.^[^ ^55^ ^]^ The catalytic cycle is completed by Trx reductase (TrxR), a selenoenzyme dependent on NADPH, which continuously regenerates the reduced, active state of Trx.^[^ ^56^ ^]^ Functionally, the Trx system detoxifies peroxides (with peroxiredoxins), modulates the activity of proteins like transcription factors (AP‐1, NF‐κB) via cysteine reduction, and inhibits apoptosis by regulating kinases such as ASK1.^[^ ^54^, ^57^ ^]^ In many cancers, the Trx system components (Trx, TrxR) are upregulated, correlating with enhanced proliferation, survival against oxidative stress, and therapy resistance.^[^ ^58^ ^]^ This dependency makes TrxR a therapeutic target, with inhibitors like auranofin explored for anticancer activity.^[^ ^59^ ^]^ However, the state of the Trx system is critical. While protective against oxidation, excessive reduction (reductive stress) can be detrimental.^[^ ^34^ ^]^ An overly reduced Trx pool might impair regulatory functions, promote pro‐inflammatory pathways like inflammasome activation,^[^ ^34^, ^60^ ^]^ or quench essential ROS signaling required for normal cell function.^[^ ^61^ ^]^ Together with GSH, the Trx system forms the core of cellular redox control. Its upregulation in cancer provides a survival edge but also establishes a precarious redox balance. Disturbing this balance, either toward excessive oxidation or potentially through excessive reduction, represents a key area of investigation in cancer biology and therapy.

Overall, the sources of reducing equivalents in cells (NADH, NADPH, GSH, Trx) are deeply intertwined with metabolism and signaling. Cancer cells recalibrate these systems to survive oxidative stress and fuel rapid growth, but this often means walking a fine line. An overshoot in any one of these reducing power sources can upset the redox equilibrium and induce reductive stress. Some cancer cells can rebalance their redox systems, whereas others, constrained by their inherent traits, cannot.

### 2.3 Selective Sensitivity of Tumors to Reductive Stress

Cancer cells differ greatly in how they respond to reductive stress, reflecting their diverse genetic makeup and metabolic profiles. Some tumors can thrive even when their internal environment becomes highly reduced, whereas others become vulnerable under these conditions. This selective sensitivity is often connected to how cancer cells handle reductive stress and their reliance on certain redox signaling pathways.^[^ ^36^, ^37^ ^]^ Key determinants of tumor sensitivity include the activity of antioxidant pathways, metabolic preferences (mitochondrial respiration vs glycolysis), ROS‐driven oncogenic signaling, and cellular processes maintaining protein balance. Clarifying these underlying factors helps identify which tumors are most likely to respond effectively to therapies targeting the redox balance.

#### 2.3.1 NRF2/KEAP1‐Mutant Tumors

Mutations hyperactivating the NRF2 pathway, often through NRF2 gain‐of‐function or KEAP1 loss‐of‐function, are prevalent in cancers like lung and liver.^[^ ^62^ ^]^ Constitutive NRF2 activation enhances antioxidant defenses by increasing GSH, NADPH, and other reducing equivalents, conferring resistance to oxidative damage and therapy‐induced ROS.^[^ ^53b^ ^]^ Although beneficial for cellular growth, this highly reducing environment introduces a vulnerability, as the excessive accumulation of reductants may exceed cellular tolerance thresholds, thereby triggering reductive stress. For example, extreme NRF2 stabilization promoted tumor initiation but inhibited later progression in a lung cancer model, indicating a detrimental threshold for hyper‐reduction.^[^ ^63^ ^]^ Identifying tumors susceptible to reductive stress requires phenotypic confirmation via metabolic/redox indicators (e.g., high GSH/GSSG or NADH/NAD^+^ ratios), as genotype alone is insufficient. Notably, such tumors may rely heavily on pathways consuming excess reducing power, like mitochondrial NADH oxidation. Inhibiting these pathways (e.g., Complex I blockade) can disrupt redox homeostasis, selectively compromising NRF2/KEAP1‐mutant cancers pushed beyond their reductive tolerance limit.^[^ ^24^ ^]^ Thus, while NRF2/KEAP1‐mutant cancers thrive on a tilted redox balance, they become selectively vulnerable if pushed past their reductive level tolerance.

#### 2.3.2 High OXPHOS‐Dependent Tumors

Some tumors rely heavily on mitochondrial OXPHOS for ATP production, showing high oxygen consumption and low glycolytic dependence.^[^ ^64^ ^]^ These cells maintain a delicate balance between NADH production and oxidation.^[^ ^65^ ^]^ If OXPHOS capacity is impaired or saturated, NADH can rapidly accumulate, driving the cell into reductive stress.^[^ ^24^ ^]^ Such tumors are acutely sensitive to interventions that disrupt redox equilibrium. A striking example was observed in certain non‐small cell lung cancer cells with high OXPHOS: acute NRF2 activation (which increases antioxidant levels) caused a buildup of NADH that potently blocked proliferation.^[^ ^24^ ^]^ In these cells, NADH accumulation was proven to be the cause of growth inhibition, as tweaking NADH production/consumption alone could replicate or rescue the effect.^[^ ^64b^ ^]^ This suggests that OXPHOS‐dependent tumors have a lower threshold for reductive stress, where even modest inhibition of NADH oxidation (e.g., with a Complex I inhibitor) can disrupt metabolism. Identifying these tumors may involve profiling metabolism (high oxygen consumption, elevated mitochondrial gene expression) and measuring redox cofactor ratios. The “tipping point” of reductive stress varies by tumor, requiring individualized assessment of NADH buffering capacity, highlighting the importance of precise redox status measurement.

#### 2.3.3 ROS‐Signaling Dependent Tumors

Certain cancers rely on moderate ROS levels, not just as byproducts, but to drive proliferation and survival signals. These tumors use ROS‐mediated pathways such as MAPK, PI3K/AKT, NF‐κB, and HIF‐1 to promote growth and metastasis.^[^ ^66^ ^]^ For example, certain leukemias like AML generate significant NADPH oxidase (NOX)‐derived ROS to stimulate cell division,^[^ ^67^ ^]^ and Ras or MYC‐driven tumors often have elevated ROS acting as second messengers.^[^ ^68^ ^]^ These cells maintain a finely tuned redox state, with enough ROS for signaling without causing damage. However, this balance is fragile. An excessively reducing environment, such as from antioxidant treatment or excess reducing agents, can disrupt it, leading to reductive stress. In this state, ROS signals are quenched, impairing vital signaling pathways and making these tumors vulnerable to growth inhibition or apoptosis. Assessing ROS‐dependent tumors involves measuring baseline ROS levels, quantifying the activity of ROS‐generating enzymes like NOX, or evaluating the activity of redox‐sensitive transcription factors.^[^ ^69^ ^]^

#### 2.3.4 Autophagy‐Deficient Tumors

Impairments in autophagy, a critical cellular recycling and stress‐response pathway, can indirectly make tumor cells susceptible to reductive stress. When autophagy is defective, the adaptor protein p62/SQSTM1, normally degraded via this process, accumulates.^[^ ^70^ ^]^ This buildup sequesters KEAP1, the negative regulator of NRF2, leading to constitutive NRF2 activation.^[^ ^70^ ^]^ Thus, autophagy‐deficient tumors often resemble NRF2/KEAP1‐mutant tumors, studies confirm that cells lose autophagy establishing a highly reduced intracellular milieu.^[^ ^71^ ^]^ This state predisposes cells to reductive stress. Additionally, the absence of effective autophagy hinders the clearance of damaged organelles and misfolded proteins.^[^ ^72^ ^]^ In a highly reduced environment, this can exacerbate proteotoxic stress, potentially through the accumulation of misfolded proteins. These tumors are often particularly sensitive to further redox perturbations because their antioxidant systems are already maximally engaged. Pushing them into an even more reduced state, or preventing the management of excess reduced molecules, can overwhelm cellular balance and trigger cell death pathways. Recognizing these autophagy‐impaired, NRF2‐elevated tumors involves detecting markers such as high p62 levels, specific LC3 autophagosome patterns, or increased NRF2 target gene expression.^[^ ^69b^ ^]^ As with NRF2‐mutant tumors, confirming the redox state through metrics like NADH/NAD^+^ ratios or GSH levels is valuable for verifying the highly reduced condition that underpins their vulnerability. Since each autophagy‐deficient tumor has a unique ability to tolerate reductive stress, it is crucial to assess their tolerance to such stress before applying therapeutic strategies aimed at inducing further reductive burden.

Exploiting reductive stress as a therapeutic strategy critically depends on the specific redox context of the individual tumor. The goal is to selectively push cancer cells beyond their tolerance limits, particularly targeting those already operating near a reductive threshold or intrinsically reliant on an altered redox balance,^[^ ^73^ ^]^ thereby inducing cell death while ideally sparing normal cells, which typically maintain more robust redox homeostasis.^[^ ^74^ ^]^ Furthermore, the “lethal threshold” for reductive stress is not universal, it varies significantly based on a tumor's specific metabolic dependencies and redox buffering capacity. A level of reducing equivalents detrimental to one tumor type might be tolerated by another.^[^ ^74^, ^75^ ^]^ Compounding this complexity, a tumor's redox state is highly dynamic and heterogeneous.^[^ ^63^, ^76^ ^]^ It can fluctuate significantly during tumor progression, vary across different locations within the primary tumor and metastases, and adapt in response to other interventions like chemotherapy.^[^ ^77^ ^]^ Identifying patients whose tumors already operate near a reductive threshold therefore demands real‐time, tumor‐specific profiling of reactive‐oxygen species, key redox couples (e.g., NADH/NAD^+^, GSH/GSSG), and antioxidant reserves before and during therapy.

## 3 The Detection of Reductive Stress

Translating the concept of targeting reductive stress into effective therapies hinges on the ability to accurately detect and measure this state. Robust detection methods are crucial for confirming the reductive environment in specific tumors, guiding the development of interventions like redox‐modulating nanomedicines, and assessing whether these treatments successfully induce the desired redox shifts. However, measuring reductive stress presents significant technical challenges. It entails capturing subtle shifts in intracellular redox couples (e.g., NADH/NAD^+^ or GSH/GSSG) often in real‐time and within specific subcellular locales.^[^ ^15^ ^]^ However, no single assay can comprehensively monitor reductive stress across all biological contexts. Therefore, researchers use a combination of approaches, including traditional biochemical assays, genetically encoded probes, and advanced imaging modalities, to gauge the reducing state of cells and tissues (**Table** **1**). Each method offers distinct advantages and limitations, and together they provide complementary windows into the redox status of a system. Notably, these detection tools also support nanotherapy development and evaluation: they allow investigators to verify whether a given nanomedicine or other intervention is inducing the intended reductive shift in the tumor environment, and to fine‐tune such therapies based on quantitative feedback. The major detection methods are categorized by technological type (classical assays, sensors, imaging techniques, and biomarkers), highlighting their principles, strengths and weaknesses, representative applications (especially in cancer research), and their relevance for assessing reductive stress treatments.

**Table 1:** Comparison of Reductive Stress Detection Methods.

Method	Mechanism	Strengths	Limitations	Typical Application
Classic biochemical assays	Enzymatic or colorimetric assays on cell/tissue lysates; HPLC/MS quantification of redox couples (e.g. GSH/GSSG, NAD(P)H/NAD(P)^+^)	High sensitivity; quantitative (absolute or ratio); well‐established protocols	Requires lysis; yields only bulk‐average results; prone to processing artifacts	Endpoint profiling of cells or tissues; validation of treatment effects
Genetically encoded sensors	Fluorescent proteins fused to redox‐sensitive domains (e.g., roGFP, SoNar, NAPstars)	Live‐cell, real‐time monitoring; organelle targeting; ratiometric signals	Requires genetic delivery; limited dynamic range; sensitive to pH and sensor expression levels	Imaging redox dynamics in cell and animal models; high‐content screening
Synthetic fluorescent probes	Small molecules that react with specific reductants (e.g. reversible Michael‐addition to GSH)	Fast response; cell‐permeable; ratiometric signals	Variable specificity and reversibility; can consume analyte; photostability issues	Live‐cell fluorescence assays; microscopy of thiols and other redox metabolites
Optical redox imaging	Label‐free NADH/FAD autofluorescence via two‐photon or FLIM	Noninvasive, label‐free metabolic imaging; high subcellular resolution	Limited tissue penetration (≈300 µm); susceptible to interference from other fluorophores; requires specialized optics	Imaging tissue metabolism (e.g., tumor biopsies, organoid models)
Clinical metabolite biomarkers	Metabolites or proteins in blood/urine (e.g., α‐HB, lactate, GDF‐15) measured via MS or immunoassay	Minimally invasive; integrates systemic redox state; amenable to multiplex biomarker panels	Indirect (multi‐factorial); not real‐time; requires clinical validation	Patient stratification; monitoring systemic redox balance; prognostic biomarker panels

### 3.1 Classic Biochemical Assays of Redox Couples

Classic biochemical assays serve as fundamental tools for quantifying key redox molecules within cell, tissue, or biofluid samples. These include measurements of the GSH redox couple (reduced GSH vs. oxidized GSSG) and pyridine nucleotides (NADH/NAD^+^ and NADPH/NADP^+^). Readouts typically rely on enzyme‐cycling systems or derivatization followed by colorimetric, fluorometric, HPLC, or mass‐spectrometric detection.^[^ ^78^ ^]^ For example, quantification of GSH and GSSG can involve adding GSH reductase and a chromogenic substrate (like DTNB or monochlorobimane) to a cell lysate, producing a color or fluorescence signal proportional to GSH levels.^[^ ^79^ ^]^ Similarly, NADH and NAD^+^ are commonly measured via enzyme cycling coupled to a reporter reaction, or by advanced techniques such as high‐performance liquid chromatography (HPLC) and mass spectrometry (MS) applied to cell extracts.^[^ ^80^ ^]^ These assays are inexpensive, highly sensitive, and capable of yielding absolute concentrations as well as metabolite ratios, making them pervasive in redox profiling of cancer cell lines and tumor biopsies.^[^ ^78b^ ^]^ Beyond solid tissues, the same chemistry can be applied to periodic blood draws or core biopsies, enabling pharmacodynamic monitoring in nanomedicine studies; a rise in tumor or plasma NADH/NAD⁺ after administration of a redox‐active nanoparticle provides biochemical proof of therapy‐induced reductive stress.

The principal drawbacks are their destructive nature and limited resolution. Because samples must be lysed or homogenized, information is averaged across heterogeneous regions and confined to a single time point, precluding real‐time or sub‐cellular analysis. Sample processing steps can also inadvertently alter redox ratios unless meticulously controlled. Consequently, while indispensable as a quantitative baseline and end‐point readout, classic assays may miss rapid, compartment‐specific redox oscillations and are therefore best complemented with live‐cell sensors or imaging probes when dynamic information is required.

### 3.2 Genetically Encoded Redox Sensors

**Figure 4:**
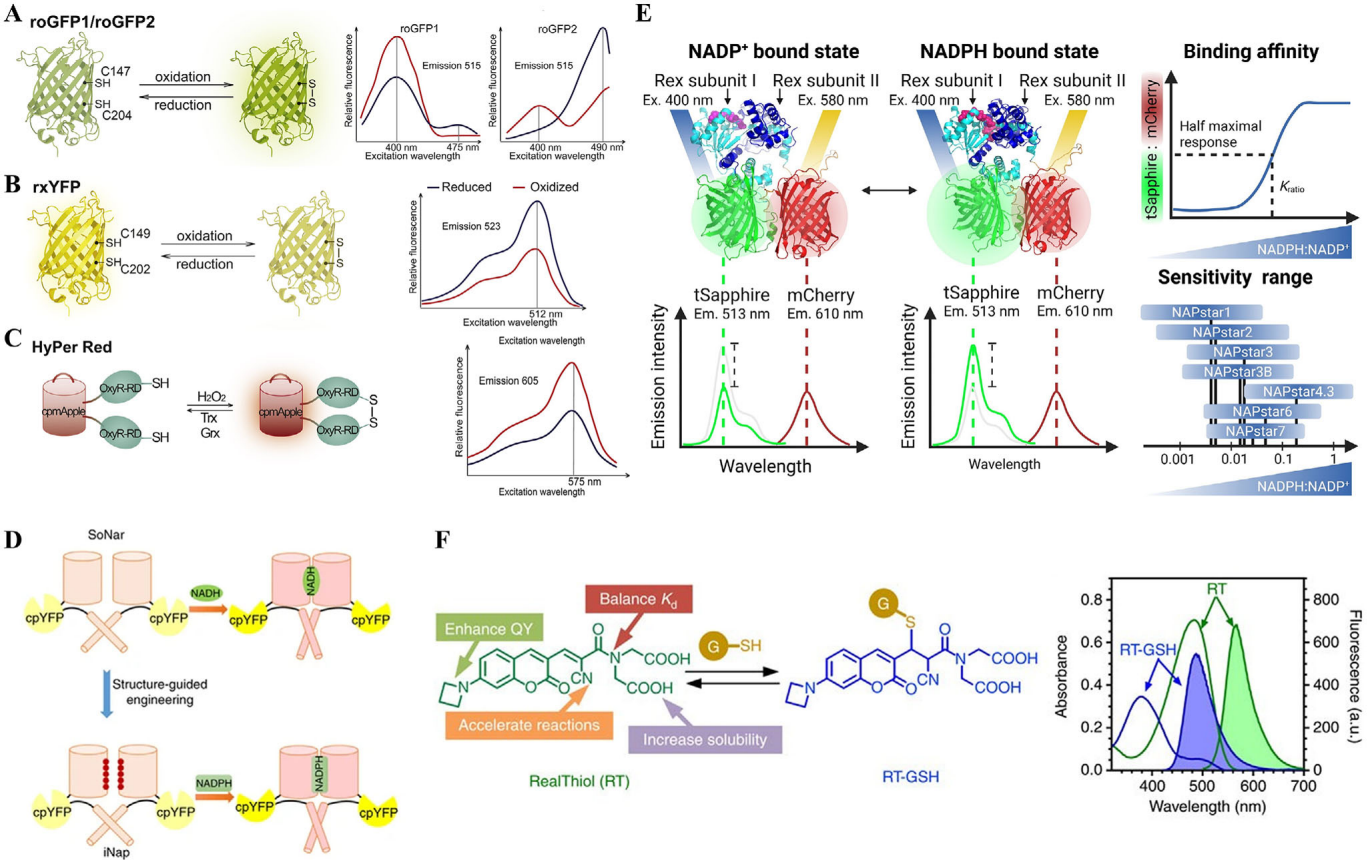
Fluorescent redox probes and their working principles. Graphical schemes and excitation spectra of indicators for protein‐based thiol sensors: A) roGFP1, roGFP2. B) rxYFP and C) HyPer Red. Reproduced with permission.^[^ ^81^ ^]^ Copyright 2016, Elsevier Inc. D) Schemes of pyridine‐nucleotide redox sensors SoNar and iNap. Reproduced with permission.^[^ ^85^ ^]^ Copyright 2017, Springer Nature E) NAPstar toggles the tSapphire/mCherry fluorescence ratio in response to NADPH‐NADP^+^ binding, and its seven variants collectively cover a detection range from 0.001 to 1 NADPH:NADP^+^. Reproduced with permission.^[^ ^86b^ ^]^ Copyright 2025, Elsevier Inc. F) RT reversibly forms a Michael adduct with glutathione (GSH), a process revealed by the corresponding shifts in its UV–vis and fluorescence spectra from RT (green) to the RT‐GSH adduct (blue). Reproduced with permission.^[^ ^89^ ^]^ Copyright 2017, Springer Nature.

Genetically encoded fluorescent sensors enable dynamic, live‐cell imaging of redox states with subcellular precision. These probes typically consist of a fluorescent protein (e.g. GFP or YFP) fused to a redox‐responsive domain that undergoes a reversible conformational change upon oxidation or reduction.^[^ ^81^ ^]^ For example, engineered redox‐sensitive GFPs (roGFP1/2 family) contain surface cysteine pairs that form a reversible disulfide under oxidizing conditions (**Figure** **4**A).^[^ ^81^ ^]^ When roGFP2 is fused to glutaredoxin‐1 (Grx1‐roGFP2), the sensor rapidly equilibrates with the cellular GSH/GSSG couple; shifts in the local glutathione redox potential appear as reciprocal changes in two excitation peaks, enabling quantitative, compartment‐specific imaging.^[^ ^82^ ^]^ By directing roGFP variants to the mitochondria, ER, and peroxisomes, or employing newer probes like rxYFP (Figure 4B),^[^ ^81^ ^]^ researchers have shown that, under stress, cancer cells maintain a highly reduced cytosolic GSH pool even as their mitochondrial or ER compartments become oxidized.^[^ ^83^ ^]^ Complementary thiol sensors like HyPer and its red‐emitting or pH‐insensitive derivatives report H_2_O_2_ dynamics and can indirectly flag reductive states when ROS levels fall below baseline expectations (Figure 4C).^[^ ^81^ ^]^ Beyond glutathione, several families track pyridine nucleotides. SoNar is a circularly permuted YFP‐based sensor that reports the cytosolic NADH/NAD⁺ ratio through NADH‐dependent modulation of dual excitation peaks (Figure 4D).^[^ ^84^ ^]^ Likewise, the iNap family was engineered from SoNar to bind NADPH selectively; iNap variants span a range of NADPH affinities (2–120 µm) suitable for typical cellular concentrations (Figure 4D).^[^ ^85^ ^]^ Most recently, the NAPstar family expanded coverage of the NADP(H) redox couple, offering tunable affinity and improved photostability for real‐time, multi‐organelle imaging of anabolic and antioxidant pathways (Figure 4E).^[^ ^86^ ^]^

The main limitations of genetically encoded sensors are practical. They require genetic delivery (transfection or stable expression) and may perturb the system. Each probe has a finite dynamic range (set by its binding affinity) and can be influenced by factors such as pH or probe expression.^[^ ^84^, ^87^ ^]^ For instance, cpYFP‐based sensors (like SoNar) are pH‐sensitive and typically require calibration controls.^[^ ^88^ ^]^ Even so, by providing ratiometric, compartment‐specific readouts, these sensors are uniquely powerful tools for studying compartmentalized redox processes in live cells and organisms.

### 3.3 Synthetic Probes and Imaging Techniques

Synthetic chemical probes provide powerful tools for imaging and monitoring cellular redox states without genetic manipulation, applicable to live cells, tissues, or entire organisms. These probes typically involve fluorescent dyes, photoacoustic (PA) contrast agents and nanoparticle‐based sensors that respond to reductants such as glutathione (GSH) or NAD(P)H. A classic fluorescent example is monochlorobimane (MCB), which forms a fluorescent adduct with GSH and has been widely used to map glutathione in cancer cells.^[^ ^90^ ^]^ NDA (naphthalene‐2,3‐dicarboxaldehyde) and OPA (o‐phthalaldehyde) likewise fluoresce upon binding NAD(P)H or GSH, enabling microfluidic redox assays.^[^ ^91^ ^]^ In contrast to irreversible reporters such as MCB, NDA, and OPA, the reversible fluorescent probe RealThiol (RT) leverages a rapid cyanoacrylamide Michael addition to quantify intracellular GSH in real time, furnishing ratiometric read‐outs within seconds in live cells and organoids and thus enabling dynamic flow‐cytometry analyses (Figure 4F).^[^ ^89^ ^]^ More advanced two‐photon dyes report GSH:GSSG ratios in tumor organoids, and a coumarin probe that shifts from blue (oxidized) to green (reduced) allows quantitative redox mapping.^[^ ^92^ ^]^ The PA dye DiOH‐BDP repeatedly reports hypochlorite (ClO^−^)/GSH cycling in liver‐injury models.^[^ ^93^ ^]^ PA imaging also offers deep‐tissue capability; Lucero et al.’s PACDx accumulates in high‐GSH tumors, and its theranostic analogue PARx releases chemotherapy while broadcasting a PA drug‐release signal,^[^ ^94^ ^]^ with new PA sensors for NADPH under development.^[^ ^95^ ^]^

Nanotechnology extends these principles by integrating sensing and delivery. Polymer nanoparticles whose fluorophores are quenched by disulfide linkages become fluorescent when intratumoral thiols cleave the bonds, visualizing payload release.^[^ ^96^ ^]^ Up‐conversion nanoparticles and quantum dots coated with redox‐sensitive layers emit near‐infrared signals only in reducing environments.^[^ ^97^ ^]^ A notable example is a polysulfide nanoparticle loaded with the PA dye CY‐PSD: reaction with tumor thiols liberates H₂S and shifts the PA spectrum, confirming selective activation within reductive tumor microenvironments.^[^ ^98^ ^]^

These synthetic probes are prized for ease of use, high sensitivity, and rapid response, making them suitable for clinical samples and animal models. Limitations include cross‐reactivity (some GSH probes also detect other thiols; NADH sensors can respond to NADPH), uneven penetration in solid tumors, and background autofluorescence, issues mitigated by careful calibration, ratiometric designs, and rigorous controls. Collectively, synthetic chemical probes and imaging techniques deepen our understanding of redox biology and offer promising routes to personalized cancer therapy, coupling drug action with real‐time, non‐invasive monitoring of treatment outcomes.

### 3.4 Clinical and Translational Redox Biomarkers

Beyond laboratory assays and imaging, there is growing interest in identifying circulating or tissue biomarkers that signal the presence of reductive stress in cancer patients.^[^ ^37^, ^74^ ^]^ These biomarkers could be small‐molecule metabolites or proteins whose levels change in response to a shift toward a more reduced redox state.^[^ ^99^ ^]^ The appeal of biomarkers is their potential for non‐invasive detection (e.g., blood tests) and use in therapeutic monitoring or patient stratification.^[^ ^100^ ^]^ If a reliable biomarker of reductive stress is found, it could indicate which patients have tumors under high reductive pressure (and might benefit from certain redox‐based therapies) or serve as an early readout of treatment response.

A critical translational challenge is identifying accessible biomarkers of reductive stress in patients. Metabolite biomarkers have shown particular promise, as their levels closely correlate with intracellular redox changes. Elevated levels of the key metabolite α‐hydroxybutyrate (α‐HB) correlate significantly with higher NADH/NAD⁺ ratios, a hallmark of reductive stress, and in both animal models and humans, high hepatic NADH drives a substantial rise in plasma α‐HB.^[^ ^101^ ^]^ Another metabolic indicator is the lactate‐to‐pyruvate ratio, which typically rises under conditions of high NADH/NAD⁺ ratios.^[^ ^102^ ^]^ Although lactate alone is not specifically indicative of reductive stress, research has identified more specific metabolites such as N‐lactoyl‐amino acids and β‐hydroxy fatty acids in conditions marked by NADH overload.^[^ ^103^ ^]^ Among protein biomarkers, Growth differentiation factor 15 (GDF15) is gaining prominence. Known as a stress‐responsive cytokine, GDF15 is notably elevated under reductive stress conditions.^[^ ^104^ ^]^ Studies have demonstrated that cells forced into a reductive state show pronounced increases in GDF15 expression.^[^ ^105^ ^]^ Elevated GDF15 has also been observed in patients with mitochondrial disorders, conditions typically associated with reductive stress.^[^ ^104^, ^106^ ^]^ These findings suggest that analogous metabolic signatures might be sought in cancer or other diseases characterized by reductive stress.

Currently, routine clinical assays do not specifically distinguish reductive from oxidative stress.^[^ ^107^ ^]^ Standard blood tests (total GSH, thiol/disulfide ratios, antioxidant enzyme activities) reflect overall redox balance but are influenced by many factors.^[^ ^108^ ^]^ Translational efforts are therefore focusing on multi‐parametric approaches: for example, combining metabolic ratios (e.g. lactate/pyruvate or plasma α‐HB), redox‐responsive proteins (e.g. elevated GDF‐15 or heme oxygenase‐1), and functional imaging surrogates (e.g. MR or PET redox probes) to report tumor redox status.^[^ ^101^, ^109^ ^]^ Such panels of metabolic and protein biomarkers may eventually enable noninvasive monitoring of reductive stress in patients, aiding risk stratification or therapy monitoring.

In summary, a variety of techniques, each with its own strengths and limitations, have been employed to monitor reductive stress levels in tumor cells. Whether through conventional biochemical assays or state‐of‐the‐art imaging sensors, these methods give us critical windows into the redox landscape of tumors. Such insight forms the foundation for precision intervention: only by accurately gauging intratumoral reductive stress can we design nanomedicine strategies that specifically target this aberrant redox state.

## 4 Nanomedicine‐Driven Modulation of Reductive Stress

Exploiting reductive stress for cancer therapy requires the precise delivery of reductive stimuli to tumor cells.^[^ ^110^ ^]^ Nanomedicine has emerged as a powerful strategy in this context, enabling targeted delivery, controlled release, and customizable nanoscale interventions. By harnessing the unique properties of nanocarriers, including the enhanced permeability and retention effect,^[^ ^111^ ^]^ surface functionalization for active targeting,^[^ ^112^ ^]^ and stimulus‐responsive payload release,^[^ ^113^ ^]^ researchers are developing approaches to selectively induce reductive stress within tumors. Nanosystems can protect sensitive redox‐active compounds from degradation during circulation and concentrate them in tumor tissue, thereby minimizing systemic toxicity. Furthermore, nanocarriers can be engineered to respond to specific triggers within the tumor microenvironment.^[^ ^112^ ^]^ For instance, they can release their payload in response to the reductant‐rich, acidic, or hypoxic conditions characteristic of tumors, or upon exposure to external stimuli like light.^[^ ^114^ ^]^ These capabilities overcome many limitations of conventional drug delivery and align well with the key principles of targeted redox therapy: delivering the right species to the right place at the right time and dose.^[^ ^6^ ^]^ Below, we highlight three main nanomedicine‐based approaches for modulating reductive stress in cancer:^[^ ^115^ ^]^ 1) nanocarriers delivering reducing agents into tumor cells; 2) nanoplatforms that generate reductive conditions in situ (e.g. via triggered release of electrons or reductive gas molecules); and 3) antioxidant nanoenzymes (nanozymes) that drive cells into a reduced state by scavenging ROS. Each strategy is discussed with representative examples, along with an evaluation of their strengths and current limitations.

### 4.1 Nanocarriers for Reducing Agents

**Figure 5:**
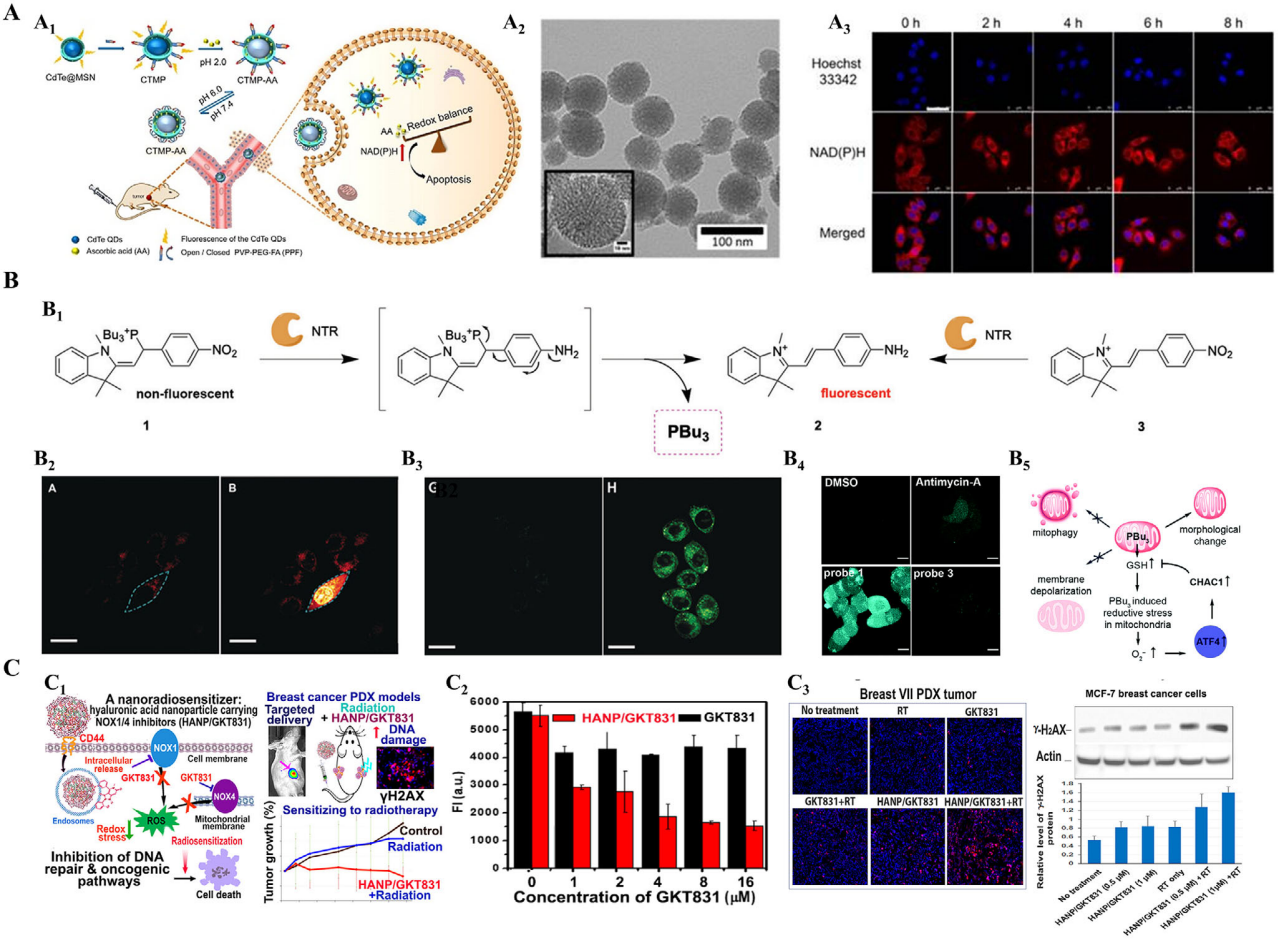
Nanocarriers with reducing agents induce reductive stress for anticancer therapy. A) pH‑responsive ascorbate nanomedicine (CTMP‑AA) induces reductive stress in HepG2 cells. A_1_) Schematic illustration of the synthesis and in vivo mechanism of the nanocarrier CTMP‐AA. A_2_) The TEM images of CdTe@MSN. A_3_) Confocal fluorescence images of NAD(P)H in HepG2 cells incubated with CTMP‐AA under hypoxia (1% O2) for different times (0, 2, 4, 6 and 8 h). Scale bar = 50 µm. Reproduced with permission.^[^ ^118^ ^]^ Copyright 2019, Ivyspring International Publisher. B) Trialkylphosphine‐based probes for inducing reductive stress. B_1_) Mechanism of enzymatic activation of probe 1 by mitochondrial NTRs, leading to the release of PBu_3_ and fluorescent reporter 2. B_2_) Single‐cell photoactivation of probe 5c shows localized fluorescence increase after FRAP (405 nm). B_3_) Photoactivation of 5c induces protein aggregation detected by increased Thioflavin T (ThT) staining. B_4_) Activation of probe 1 increases mitochondrial superoxide (O_2_⁻), similar to antimycin A (Am‐A), unlike control probe 3. Scale bar = 10 µm. B_5_) Model summarizing mitochondrial responses to reductive stress. Reproduced with permission.^[^ ^119b^ ^]^ Copyright 2016, Wiley‐VCH. Reproduced with permission.^[^ ^120b^ ^]^ Copyright 2021, The Royal Society of Chemistry. C) CD44‑targeted hyaluronic‑acid nanocarriers of NOX1/4 inhibitor GKT831 amplify reductive stress to radiosensitize breast‑cancer xenografts. C_1_) Illustration of the mechanism of action and therapeutic efficacy of the HANP/GKT831 nanoplatform. C_2_) Flow cytometry analysis showing intracellular ROS levels in MCF‐7 cells after treatment. C_3_) γ‑H2AX immunofluorescence of breast‑PDX sections and accompanying western blot of DNA‑damage markers. Reproduced with permission.^[^ ^121^ ^]^ Copyright 2022, American Chemical Society.

A primary strategy involves using nanoparticles as carriers for small‐molecule reductants, such as antioxidants or other reducing agents, to flood tumor cells with reducing equivalents. The rationale is that tumors often exhibit elevated baseline levels of reductive species like GSH and NAD(P)H^[^ ^116^ ^]^; delivering an additional surge of reductants may overwhelm their redox buffering systems, thereby triggering cytotoxic reductive stress. This effect is expected to be relatively tumor‐selective, as normal cells are typically better equipped to manage elevated reductive conditions. Preclinical studies confirm that certain reductive molecules can selectively eliminate cancer cells. For example, while high‐dose ascorbic acid (vitamin C) can act as a pro‐oxidant at pharmacological concentrations, generating lethal ROS,^[^ ^117^ ^]^ evidence suggests its reducing properties might also be harnessed therapeutically if delivered appropriately. The main challenges are ascorbate's poor cellular uptake and rapid metabolism. To address this, Gao et al. designed a nanoparticle (CTMP‐AA) that carries ascorbic acid in a tumor‐targeted, pH‐responsive formulation (**Figure** **5**A).^[^ ^118^ ^]^ This nanoparticle featured a CdTe quantum dot core within a mesoporous silica shell, coated with poly‐2‐vinylpyridine and folic acid for targeting hepatocellular carcinoma cells. Within the hypoxic, acidic tumor environment, CTMP‐AA released ascorbate intracellularly, significantly increasing NADPH levels and inducing reductive stress in cancer cells. This disruption of redox balance activated apoptotic pathways, leading to significant tumor cell death in vitro and suppressed tumor growth in vivo, with minimal harm to normal cells, indicating a favorable therapeutic window. This example illustrates how a nanocarrier can deliver a conventional “antioxidant” in a manner that paradoxically triggers lethal reductive stress in cancer.

Beyond vitamins, synthetic reductants such as trialkylphosphines, which are potent agents specifically reducing disulfide bonds like GSSG to GSH with minimal off‐target effects, have been utilized in the design of cell‐permeable molecular probes.^[^ ^119^ ^]^ Nguyen and colleagues developed trialkylphosphine‐based probes that are activated either enzymatically in mitochondria or photochemically in the cytosol (Figure 5B).^[^ ^119^, ^120^ ^]^ These tools allowed researchers to induce localized reductive stress and monitor cellular responses. Hallmarks of this stress included protein aggregation, a classical indicator of reductive imbalance. Intriguingly, these reductive conditions also led to significant accumulation of superoxide radicals (O_2_^•−^), especially in mitochondria. This paradoxical finding suggests that an excessively reduced environment may enhance electron leakage from the respiratory chain, promoting ROS formation and amplifying cellular damage. The activation of the ATF4‐CHAC1 signaling pathway and mitochondrial morphological changes, in the absence of membrane depolarization or mitophagy, further illustrate this complex redox crosstalk.

In addition to delivering reductive agents, the delivery of drugs that can indirectly regulate the intracellular redox state also constitutes a feasible strategy. Tumors often rely on a baseline of ROS signaling; stripping this away can weaken cancer cell defenses or even directly induce a form of reductive stress. For example, an innovative study encapsulated a NOX inhibitor in CD44‐targeted hyaluronic acid nanoparticles (HANP/GKT831) to shut down a major source of ROS in cancer cells (Figure 5C).^[^ ^121^ ^]^ By inhibiting NOX1 and NOX4 enzymes, this nanomedicine drastically reduced intracellular ROS generation, effectively forcing the tumors into a more reduced state in patient‐derived breast tumor xenografts. The result was not only tumor growth inhibition on its own but also a markedly improved response to subsequent radiotherapy.

A significant advantage of reductant‐delivering nanocarriers is their relative simplicity and versatility; various antioxidants or thiol donors, such as N‐acetylcysteine or GSH esters, can be effectively packaged into tumor‐targeted nanoparticles. Precise control over release kinetics is crucial to avoid systemic side effects or inadvertently protecting the tumor, as achieving an optimal level of reductive stress is critical; insufficient stress may allow tumor adaptation, while excessive or rapid delivery could harm adjacent normal tissues. Encouragingly, preclinical studies have shown that these nanomedicines can effectively leverage the tumor's own antioxidant milieu to selectively induce lethal reductive stress, with early in vivo studies indicating a favorable therapeutic window. Although promising, most reductant‐carrying nanomedicines are still undergoing preclinical optimization and require comprehensive long‐term safety assessments before clinical translation.

### 4.2 In Situ Induction of Reductive Stress

**Figure 6:**
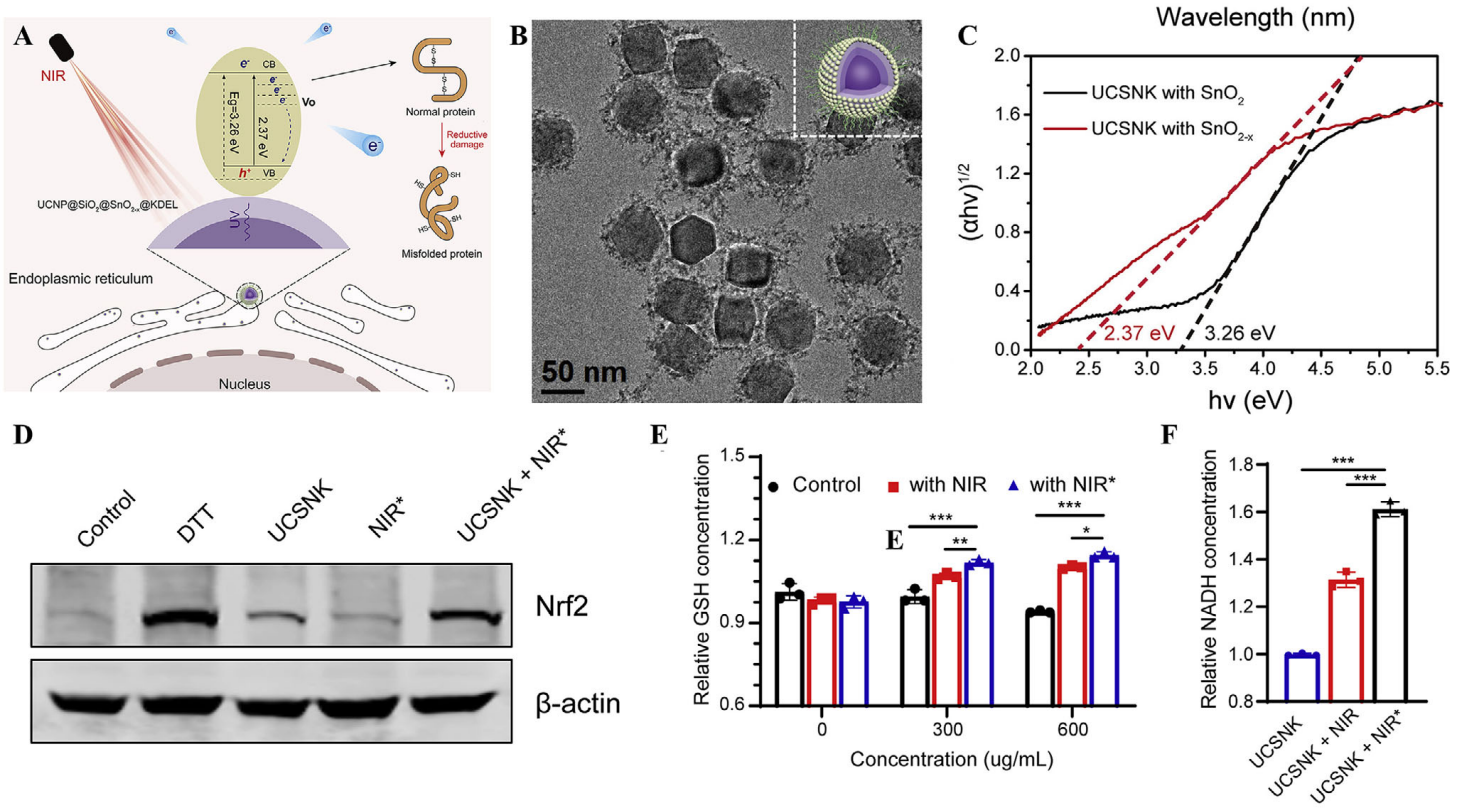
ER‐targeted reductive stress induced by NIR‐activated UCSNK nanoparticles. A) Schematic illustration: 980 nm NIR light is converted by Yb^3+^/Tm^3+^‐doped UCNPs into UV/visible photons that excite an oxygen‑deficient SnO_2‐x_ shell, releasing photo‑generated electrons that selectively reduce disulfide bonds of ER proteins, causing misfolding, activation of the UPR and ER‑stress‑mediated apoptosis. B) TEM image shows the uniform hexagonal core‐shell architecture of UCSNK; the inset depicts the UCNP core and SnO_2‐x_ shell. C) Tauc plots reveal a narrowed band gap of 2.37 eV for the SnO_2‐x_ shell (red) versus 3.26 eV for stoichiometric SnO_2_ (black). D) Western blot of Nrf2 in 4T1 cells after the indicated treatments. E) Relative intracellular GSH levels at increasing UCSNK doses under irradiation at 980 nm. F) Relative NADH levels highlight the greatest reductive shift in the UCSNK + NIR^*^ group. NIR^*^ represents 10 min irradiation at 980 nm (1 W cm^−2^). Reproduced with permission.^[^ ^7^ ^]^ Copyright 2022, Elsevier Inc.

A more ingenious approach goes beyond carrying a reductive payload and instead uses nanoplatforms that generate a reductive stimulus in situ within the tumor. In other words, rather than loading a nanoparticle with a reducing agent, these strategies induce reductive conditions on demand, often leveraging external triggers or unique tumor microenvironment (TME) features to activate the effect. In 2016, we designed an acid‐activated Mg_2_Si nano “deoxygenator” that, once inside a tumor, rapidly liberates the highly reductive agent silane (SiH_4_), enabling efficient local oxygen scavenging through vigorous redox reactions, while simultaneously generating SiO_2_ micro‐emboli that block the blood supply, thereby plunging the tumor into extreme hypoxia and a reductive state.^[^ ^122^ ^]^ This work inspired us to explore the potential of leveraging reductive stress in cancer therapy. Building on this premise, our group proposed an innovative cancer treatment strategy known as “electron interference therapy” (**Figure** **6**) in 2022.^[^ ^7^ ^]^ We developed UCNP@SnO2‐x@KDEL nanoparticles (UCSNK) that couple tissue‐penetrating near‐infrared (NIR) activation with endoplasmic‐reticulum‐targeted electron delivery to intensify reductive stress in a spatially and temporally controlled manner. Specifically, the core consists of Yb^3+^/Tm^3+^‐doped upconversion nanoparticles (UCNPs) that convert NIR light to localized UV/visible emissions (Figure 6A).^[^ ^7^ ^]^ These photons in turn photo‐excite a sub‐stoichiometric SnO_2‐x_ semiconductor shell (Figure 6B,C), liberating a burst of high‐energy electrons. Additionally, surface conjugation with KDEL peptides directs the entire construct to the ER, ensuring that the photogenerated electrons are released at the site where disulfide‐rich proteins undergo folding. This disrupts protein structures and causes extensive protein misfolding, triggering intracellular reductive stress (Figure 6D,E), activating the UPR, and ultimately inducing ER stress‐mediated apoptosis. The study demonstrates, through both in vitro and in vivo models that NIR‐triggered UCSNK can effectively induce tumor cell death and significantly inhibit tumor growth while causing minimal damage to surrounding healthy tissue (since the therapeutic effect is only activated in the irradiated region). This method uniquely utilizes reductive stress, in contrast to the traditional oxidative stress, as an anticancer strategy, offering precise spatial and temporal control while minimizing systemic side effects during treatment. However, this strategy requires external NIR light activation, and its clinical application will depend on overcoming challenges in the delivery and control of that light.

**Figure 7:**
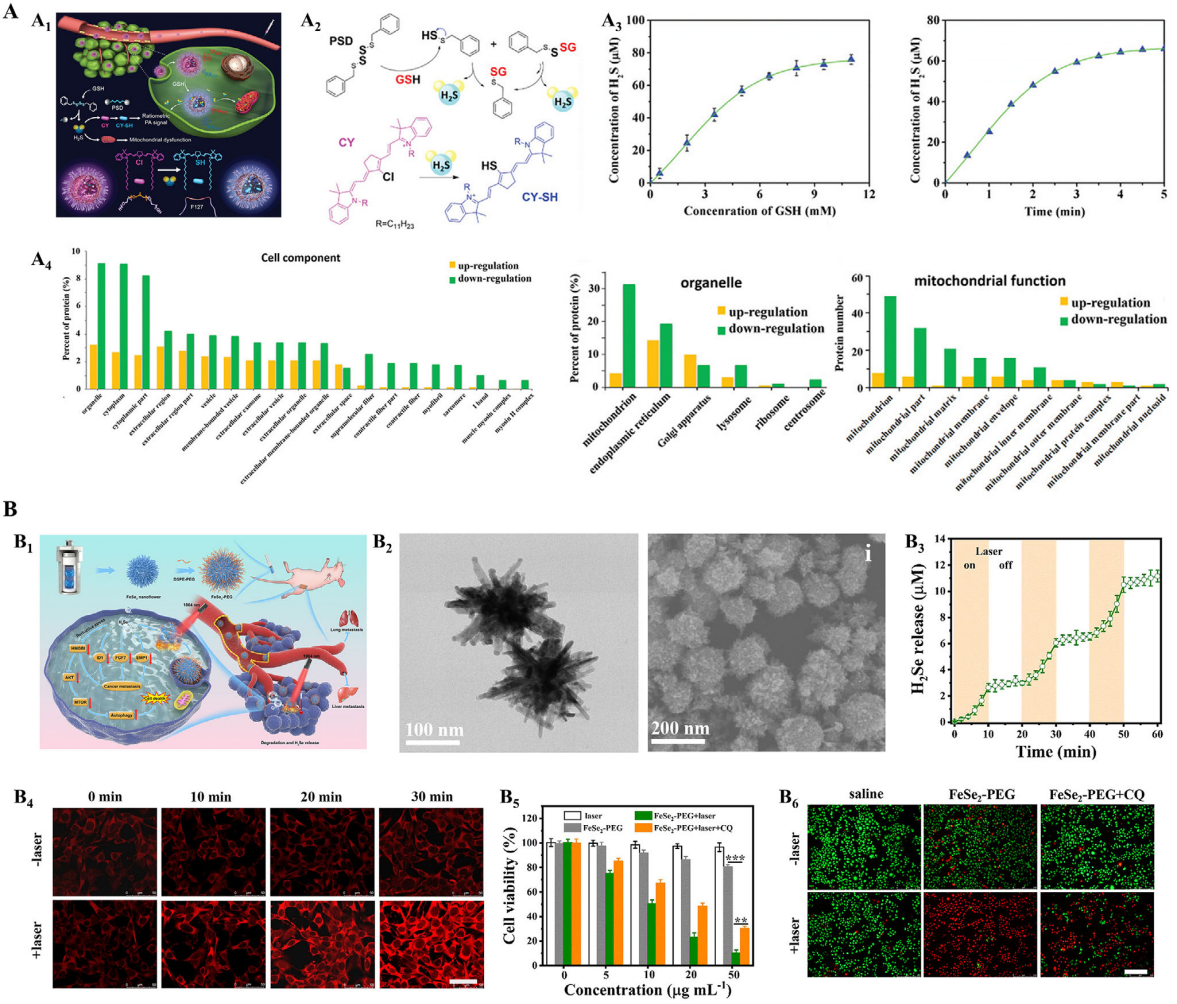
Tumour microenvironment responsive H_2_S/H_2_Se gas nanomedicines and their anticancer mechanisms. A) Polysulfide‐based (CY‐PSD) nanotheranostic system for controlled H₂S release and therapy. A_1_) Schematic of CY‑PSD nanoparticles. A_2_) Reaction pathway: thiol‐polysulfide exchange splits the PSD prodrug to emit H_2_S while converting the cyanine precursor (CY‑Cl) to the fluorescent/PA‑active CY‑SH. A_3_) Kinetics of gas release. A_4_) GO analysis of differentially expressed proteins after CY‑PSD treatment. Reproduced with permission.^[^ ^98^ ^]^ Copyright 2020, Wiley‐VCH. B) NIR‑II photoactivatable H_2_Se nanogenerator (FeSe_2_‑PEG). B_1_) Schematic of tumor‑accumulating PEGylated FeSe_2_ nanoflowers. B_2_) Electron micrographs of FeSe2 nanoflowers. TEM (left, scale bar = 100 nm) and SEM (right, scale bar = 200 nm). B_3_) On‐off 1064 nm laser (0.8 W cm^−2^) enables stepwise, on‑demand H_2_Se liberation, quantified with an H_2_Se‑selective probe. B_4_) Confocal fluorescence of 4T1 cells under hypoxia (1% O_2_). Scale bar = 100 µm. B_5_) CCK‐8 assay of 4T1 cell viability after treatments with FeSe_2_‑PEG ± laser and/or chloroquine (CQ). B_6_) Live/dead staining (Calcein‑AM/PI) corroborating enhanced cytotoxicity of FeSe_2_‑PEG + laser and its partial reversal by CQ; green = live, red = dead. Scale bars = 100 µm. Reproduced with permission.^[^ ^124^ ^]^ Copyright 2021, Elsevier.

Another set of in situ generation strategies involves nanocarriers that produce reducing gas molecules (such as hydrogen H_2_, hydrogen sulfide H_2_S, or hydrogen selenide H_2_Se) at the tumor site. These gases are known to modulate redox signaling and can induce cell death pathways when delivered at high concentrations. Hydrogen gas (H_2_), for instance, can act as a selective antioxidant by neutralizing hydroxyl radicals and peroxynitrite, and it has been explored for its anti‐inflammatory and anti‐apoptotic effects in normal tissues.^[^ ^123^ ^]^ In tumors, H_2_ might help tip the redox balance by quenching certain ROS. Hydrogen sulfide (H_2_S), on the other hand, is a reductant that can sulfhydrate protein thiols, affecting signal transduction; at elevated levels, H_2_S is cytotoxic and has been shown to induce apoptosis and even pyroptosis (inflammatory cell death) in cancer cells. Hydrogen selenide (H_2_Se) is less studied but is highly reactive and can impose reductive stress, particularly in hypoxic tumor regions, leading to inhibited tumor growth and metastasis. The challenge with therapeutic gases is controlling their delivery, as they are diffuse and can easily dissipate before reaching the target cells. Nanotechnology offers solutions via gas‐releasing nanocarriers that carry stable precursors of these gases and then activate them to release them in tumors. For instance, Li et al. engineered a polysulfide‐based nanoparticle capable of delivering hydrogen sulfide (H_2_S) in a controlled manner (**Figure** **7**A).^[^ ^98^ ^]^ Their nanoparticle, termed CY‐PSD, encapsulated an H_2_S prodrug (a compound that decomposes to produce H_2_S) along with a cyanine dye for photoacoustic imaging. The design capitalized on tumor hallmarks: in the reductant‐rich, a cysteine‐heavy environment of tumors, the polysulfide was cleaved to release H_2_S, and this process could be monitored in real‐time by the changes in the photoacoustic signal of the dye. The localized H_2_S gas burst in the tumor tissue led to mitochondrial dysfunction and triggered apoptosis in a model of triple‐negative breast cancer, significantly slowing tumor growth while causing negligible systemic toxicity. In another study, Peng et al. created FeSe_2_ nanoflowers that act as a depot for H_2_Se release upon NIR laser irradiation (Figure 7B).^[^ ^124^ ^]^ These flower‐shaped nanostructures not only released reductive H_2_Se gas under light stimulation but also generated mild heat (photothermal effect). The combination of H_2_Se‐induced reductive stress and photothermal therapy synergistically killed cancer cells and was shown to markedly suppress both primary tumor growth and metastasis in animal models.

**Figure 8:**
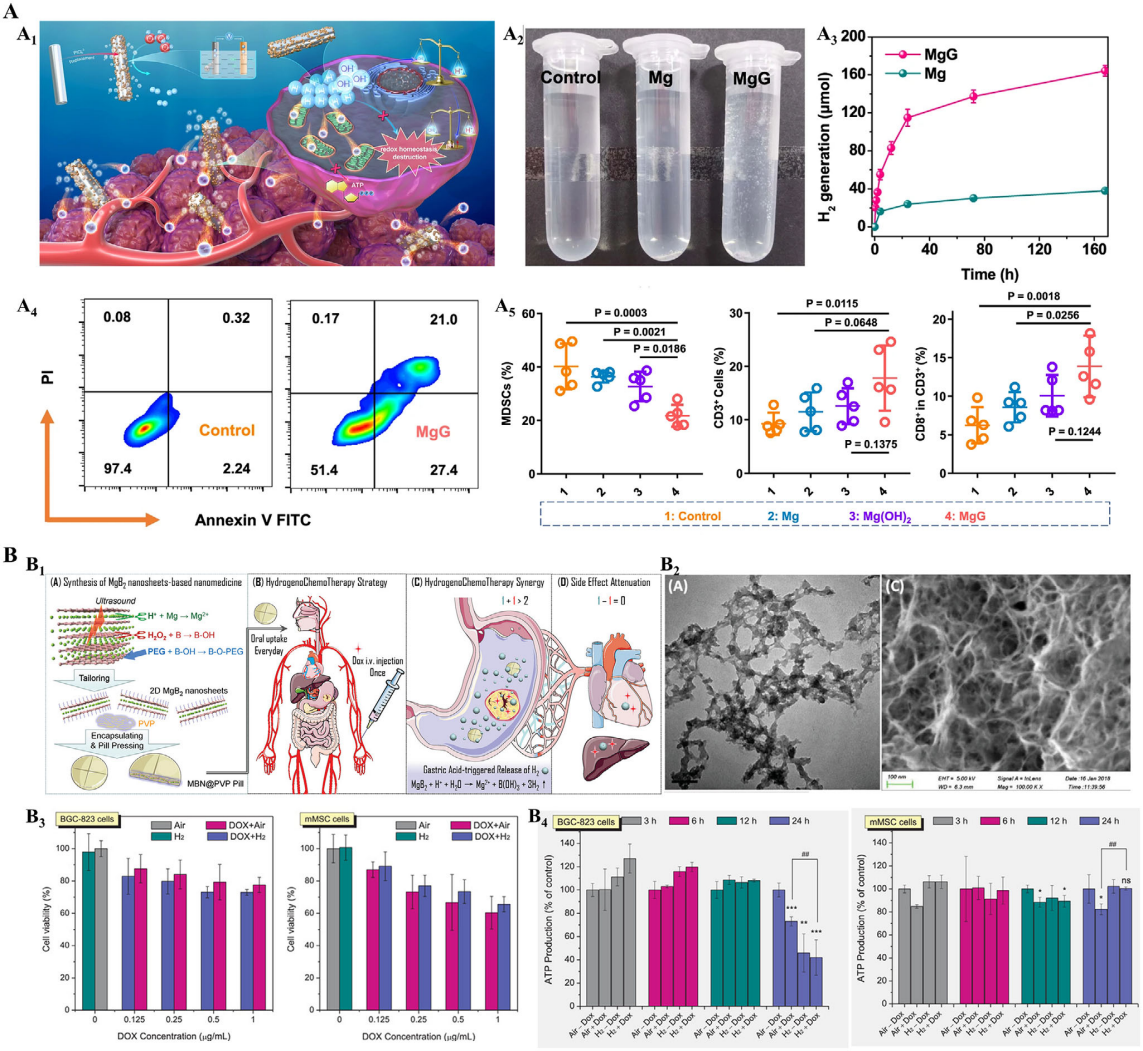
H_2_ generators drive reductive stress for tumor ablation and therapy modulation. A) Implantable MgG microbattery drives immuno‑reductive tumor control. A_1_) Schematic illustration to show Mg galvanic cell (MgG) for tumor microenvironment modulation and enhanced cancer hydrogen therapy. A_2_) Photograph showing H_2_ generation in PBS from Mg and MgG. A_3_) Time‐dependent H_2_ generation measured by gas chromatography from Mg or MgG rods in PBS (pH = 6.5, n = 3 biologically independent samples). A_4_) Annexin V‑FITC/PI flow‑cytometry plots of 4T1 cells reveal a sharp rise in early/late apoptosis after MgG treatment versus control. A_5_) Immunophenotyping of excised tumors. Reproduced with permission.^[^ ^125^ ^]^ Copyright 2022, Springer Nature. B) Acid‑triggered MgB_2_ pill boosts chemo‑reductive synergy. B_1_) Schematic illustration of the MBN@PVP pill enhances tumor killing and attenuates systemic toxicity. B_2_) TEM (left) and SEM (right) of the nanosheets. Scale bar = 100 nm. B_3_) Cell‑viability assays versus DOX concentration. B_4_) ATP‑production assays at 6, 12, and 24 h reproduce the bidirectional effect. Reproduced with permission.^[^ ^75^ ^]^ Copyright 2019, Wiley‐VCH.

One innovative tactic involves a magnesium‐based micro‐battery (MgG) developed by Yang et al. (**Figure** **8**A).^[^ ^125^ ^]^ A Mg rod coated with platinum continuously produces H_2_ when implanted in aqueous tumor tissue. The released hydrogen disrupts mitochondrial function and redox homeostasis, inducing apoptosis, while the Mg(OH)_2_ by‐product neutralizes tumor acidity, and improves the immune milieu by enhancing CD8^+^ T‐cell infiltration and reducing immunosuppressive cells. Together, these effects significantly restrained tumor progression in their model, with no notable toxicity observed. Complementary to this implantable micro‑battery paradigm, He et al. developed a non‑invasive yet equally hydrogen‑centric tactic (Figure 8B).^[^ ^75^ ^]^ They engineered polyvinylpyrrolidone‐encapsulated 2D magnesium boride nanosheets (MBN@PVP), which remain inert within neutral tissues but, upon ingestion, react with gastric acidity to gradually release H_2_ in situ. The diffusing hydrogen reproduces the microbattery mechanism by silencing mitochondrial respiration in gastric cancer cells while paradoxically boosting normal cellular metabolism; this dual effect increases the anticancer efficacy of doxorubicin while simultaneously reducing its systemic toxicity. This oral hydrogen prodrug therefore extends H_2_‑augmented therapy from surgically implanted devices to patient‑friendly pills and highlights molecular hydrogen as both a cytotoxic amplifier and a cytoprotective shield.

These examples highlight a trend toward multifunctional nanoplatforms that integrate diagnostic and therapeutic functions, often termed “theranostic” agents. In situ, the generation of reductive stress is potent because it effectively uses the tumor as a reaction vessel to produce toxic levels of reductants or electrons from relatively innocuous precursors or triggers. Nevertheless, translating these approaches to the clinic will require addressing several issues. The short half‐life and rapid diffusion of small reactive species like H_2_S or H_2_Se mean that timing and localization of release are crucial; nanocarriers must accumulate well in tumors and release their payload quickly once activated, to achieve sufficient concentration. For externally triggered systems, ensuring uniform trigger delivery (e.g., light penetration throughout a tumor mass) is an engineering hurdle. Future designs might incorporate auto‐activation mechanisms that respond to intrinsic tumor signals (such as enzyme overexpression or a specific redox threshold) to initiate gas release, reducing reliance on external triggers.

### 4.3 Antioxidant Nanoenzymes

**Figure 9:**
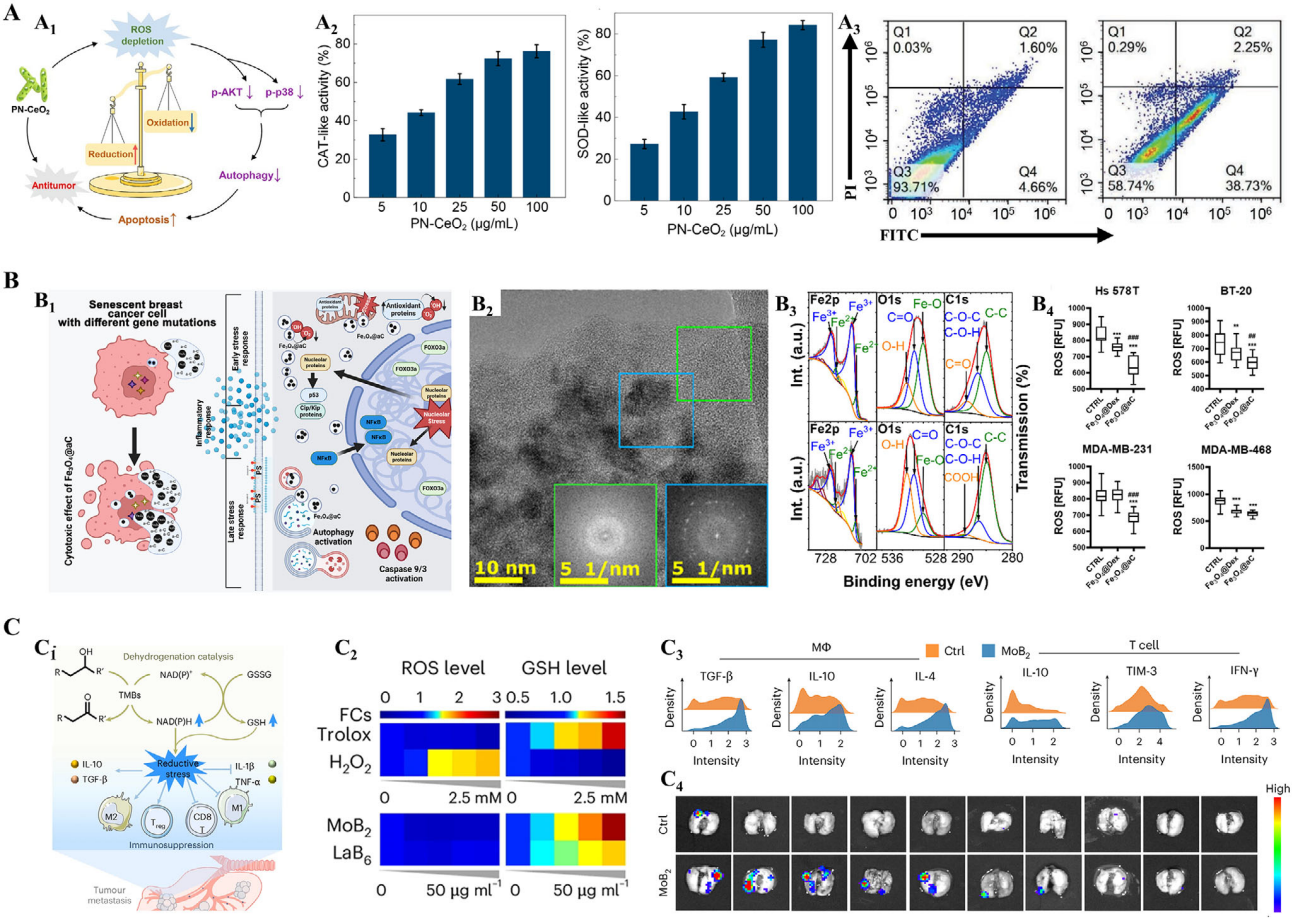
Antioxidant nanozymes drive reductive stress. A) Reductive stress therapy using porous nanoceria (PN‑CeO_2_). A_1_) Reductive‐damage strategy induced autophagy inhibition for tumor therapy. A_2_) ROS‐scavenging activities of PN‐CeO_2_. A_3_) Annexin V‑FITC/PI flow cytometry plots. Reproduced with permission.^[^ ^32^ ^]^ Copyright 2022, Tsinghua University Press. B) Fe_3_O_4_@aC induces reductive stress‐related autophagic cell death in senescent breast cancer. B_1_) Fe_3_O_4_@aC induces cytotoxicity in drug‐induced senescent breast cancer cells through reductive stress. B_2_) TEM reveals spinel‑phase Fe_3_O_4_ nanocrystals (≈8 nm) with clear lattice fringes dispersed in an amorphous carbon matrix. B_3_) High‑resolution C 1s XPS spectra of Fe_3_O_4_@aC. B_4_) Across drug‑induced senescent breast‑cancer lines a 4 h treatment (100 µg mL^−1^) significantly lowers intracellular ROS. Reproduced with permission.^[^ ^127^ ^]^ Copyright 2024, American Chemical Society. C) MoB_2_ Induced Reductive Stress Triggers Immunosuppression and Metastasis. C_1_) MoB_2_ mimics dehydrogenase activity. C_2_) A heatmap presenting the effects of MoB_2_ on cellular redox homeostasis. C_3_) CyTOF density plots show the effects of MoB_2_‐induced reductive stress on immune cell markers. C_4_) IVIS bioluminescence imaging on day 16 shows higher pulmonary photon flux in mice given three oropharyngeal doses of 2.5 mg kg^−1^ MoB_2_. Reproduced with permission.^[^ ^128^ ^]^ Copyright 2025, Springer Nature.

An alternative paradigm for inducing reductive stress in cancer cells is to remove oxidants rather than add reductants. By aggressively scavenging ROS within tumor cells, the intracellular environment can be forced into an abnormally reduced state. This strategy is implemented using antioxidant nanoenzymes, or nanozymes‐catalytic nanoparticles that mimic the activity of natural antioxidant enzymes (like SOD, catalase, or GSH peroxidase).^[^ ^126^ ^]^ Nanozymes offer advantages over protein enzymes in that they are typically more stable, can have their activity tuned by composition, and are not easily depleted. Wang et al. recently demonstrated a reductive‐damage strategy using porous cerium oxide nanorods (PN‐CeO_2_) as a redox‐active nanozyme to inhibit protective autophagy in tumor cells (**Figure** **9**A).^[^ ^32^ ^]^ Owing to their high surface area and abundant oxygen vacancies, PN‐CeO_2_ nanoparticles exhibited strong SOD‐ and catalase‐mimetic activity, catalytically converting superoxide anions to hydrogen peroxide and further decomposing hydrogen peroxide into water and oxygen. This robust ROS‐scavenging capacity led to a pronounced reductive shift in the intracellular redox environment of cutaneous squamous cell carcinoma (cSCC) cells, effectively depleting baseline ROS levels. As a result, key pro‐survival signaling pathways, including PI3K/Akt and p38 MAPK, which rely on oxidative signaling, were significantly inhibited. Concomitantly, autophagic flux was disrupted, as evidenced by reduced expression of autophagy markers Beclin1 and LC3B, along with the accumulation of phosphorylated p62 (p‐p62), indicating failure in autophagosome formation and turnover. With both antioxidant homeostasis and self‐repair mechanisms compromised, tumor cells undergo apoptosis. This work highlights reductive regulation as a promising paradigm in nanozyme‐based cancer therapy, offering a distinct mechanism from traditional ROS‐amplifying strategies. Lewińska et al. employed carbon‐coated iron oxide nanoparticles (Fe_3_O_4_@aC) as a redox‐active nanozyme platform to selectively target drug‐induced senescent breast cancer cells (Figure 9B).^[^ ^127^ ^]^ Unlike traditional iron oxide nanoparticles that often enhance ROS through Fenton‐like reactions, the amorphous carbon shell endowed Fe_3_O_4_@aC with potent ROS‐scavenging properties due to its rich content of oxygen‐containing functional groups such as hydroxyl, epoxy, and carbonyl moieties. Upon uptake by senescent tumor cells, Fe_3_O_4_@aC nanoparticles significantly reduced intracellular ROS levels and triggered a strong reductive shift in cellular redox balance. This reductive stress state initiated a cascade of downstream events: upregulation of antioxidant defenses (FOXO3a, SOD1, GPX4), activation of nucleolar and ER stress, and the induction of cytotoxic autophagy, as evidenced by increased LC3B and Beclin1 expression, coupled with nucleolar disorganization. Notably, this was distinct from the typical protective role of autophagy; here, the stress‐induced autophagic response appeared maladaptive and culminated in apoptotic cell death.^[^ ^127^ ^]^

However, not all forms of reductive stress are beneficial for cancer therapy. Extreme caution is warranted because the effects of deep redox modulation can extend beyond tumor cells alone. For example, a recent study found that using a transition‐metal boride nanozyme (molybdenum boride, MoB_2_) to induce reductive stress had unintended pro‐tumoral effects on the immune microenvironment (Figure 9C).^[^ ^128^ ^]^ In that work, MoB_2_ nanozymes potently scavenged ROS in the tumor, but this led to an immunosuppressive milieu: macrophages in the treated tumors polarized toward the M2 phenotype (immunosuppressive, pro‐tumor) and away from the tumor‐fighting M1 phenotype. Correspondingly, the levels of anti‐inflammatory cytokines (like IL‐10 and TGF‐β) increased, while pro‐inflammatory cytokines (like TNF‐α, and IL‐1β) did not rise significantly. As a result of dampening the anti‐tumor immune response, the MoB_2_‐induced reductive environment actually facilitated cancer metastasis in a breast cancer model‐mice treated with the nanozyme showing increased tumor cell spread to the lungs. This cautionary finding underscores that reductive stress therapy if not properly controlled, could inadvertently shield the tumor from immune attack or trigger other adverse pathways. It highlights the need for thorough mechanistic studies and precise targeting: for instance, strategies might be needed to confine the reductive stimulus to cancer cells while sparing immune cells or to combine reductive stress induction with immune‐modulatory treatments to prevent an immunosuppressive outcome.

Despite such challenges, antioxidant nanozymes remain attractive due to some key advantages. Because the nanozyme itself is catalytic and not consumed in the reaction, a single dose can have a sustained effect on the tumor, continuously driving down ROS levels for an extended period.^[^ ^126^, ^129^ ^]^ Many nanozymes (like cerium oxide) are also relatively biocompatible and can be engineered with targeting ligands or stimulus‐responsive coatings to improve their specificity. The versatility of nanocatalysts, in terms of material composition, tunable activity, and functionalization, provides a broad design space for future interventions focused on modulating cellular redox states in a controlled manner.

In summary, nanomedicine strategies for inducing reductive stress in cancer can be grouped into three broad categories: delivering excess reductants, generating reductive conditions on‐site (via electrons or gas molecules), and catalytically removing oxidants. Each has shown promise in preclinical models, and each comes with its own set of challenges to address. These innovative approaches open up a new front in cancer therapy: selectively driving tumor cells into lethal reductive stress or sensitizing them to other treatments.

## 5 Conclusion and Perspective

Reductive stress is increasingly recognized as a critical factor in cancer biology, presenting both challenges and opportunities for therapy. While this concept lagged for years behind the fervent study of oxidative stress, it is now clear that an excess of reductants can be as disruptive to cells as an excess of ROS. In the context of cancer, reductive stress embodies a delicate balance: tumor cells rely on heightened antioxidant systems to survive their hostile, ROS‐rich environment, yet pushing these systems beyond their limit can trigger cell death. The emerging strategies discussed in this review all seek to exploit this vulnerability in cancer cells’ redox balance by exploiting this vulnerability. Nanomedicine has been at the forefront of these efforts, enabling precise spatiotemporal modulation of tumor redox states. By selectively delivering reducing agents or ROS‐scavenging catalysts to tumors, nanocarriers achieve levels of targeting and control unattainable with conventional drugs. Importantly, they can direct therapy to specific subcellular organelles like mitochondria or the ER (where redox imbalances have maximal impact on cell fate) and can release their payloads or become activated in synchrony with external triggers or intrinsic tumor cues. This precision is especially crucial given the heterogeneity of the tumor microenvironment (e.g. hypoxic vs. normoxic regions within the same tumor),^[^ ^130^ ^]^ a globally administered redox‐modulating drug might be ineffective or even harmful in parts of the tumor or normal tissue, whereas a nanomedicine‐based approach allows for compartmentalized redox modulation.^[^ ^131^ ^]^ Such control could help overcome major barriers like drug resistance and poor therapeutic index that hinder standard cancer treatments.^[^ ^131^ ^]^

Despite notable advancements, the field of nanomedicine‐based reductive stress therapy is still in its infancy and faces many challenges. A primary concern is the quantification and optimization of the reductive stress dose needed for therapeutic efficacy. Insufficient induction of reductive stress may simply trigger adaptive responses in cancer cells (e.g., further upregulation of antioxidants or metabolic rerouting) and leave them alive and potentially more resistant. Conversely, excessive or mislocalized reductive stress could cause unintended damage, such as injuring normal cells, promoting an immunosuppressive microenvironment, or causing inflammation. Real‐time redox monitoring tools, like those described in the detection section, will be invaluable for calibrating therapies, enabling clinicians to know if a nano therapy can push a tumor's redox state into the desired range and adjust dosage accordingly. Another key issue is how to integrate reductive stress modulation with existing cancer treatments. The potential for synergy is significant. For instance, inducing reductive stress within a tumor could make it more susceptible to chemotherapy, radiation, or immune cell attack by stripping away some of its defenses (such as antioxidant buffering or autophagy). However, executing such combination strategies requires meticulous timing and sequencing, underscoring the importance of temporal control (the “Right time” aspect of the 5R principles).^[^ ^6^ ^]^ Achieving this in practice may demand smart delivery systems that can be turned on and off or multi‐component treatments where, for example, a nanocarrier releases an agent that causes reductive stress and then, after a set delay, another agent that causes oxidative stress.

From a translational perspective, the development of advanced nanocarriers remains a priority. Next‐generation nanomedicines need to demonstrate improved stability in circulation (to reach tumors more effectively), the ability to deeply penetrate tumor tissue (possibly by being small or deformable enough to navigate the dense tumor stroma), and selective uptake by cancer cells over healthy cells. Accomplishing this may involve designing smaller, more biocompatible nanoparticles, or functionalizing their surfaces with targeting moieties like peptides, antibodies, or aptamers that recognize tumor‐specific markers.^[^ ^131^, ^132^ ^]^ Additionally, considerations of biodegradability and long‐term safety of the nanomaterials are paramount for clinical translation. Any persistence of nanoparticles in the body could lead to chronic toxicity, so materials that break down into harmless byproducts are preferred.^[^ ^131^, ^133^ ^]^ Regulatory and logistical aspects will also influence the path forward. Therapies that involve external physical triggers (such as magnetic fields, light for upconversion nanoparticles, or ultrasound) require specialized equipment and protocols, which regulators will assess for safety and consistency.^[^ ^134^ ^]^ Likewise, the use of unconventional therapeutic agents like H_2_S or H_2_Se will necessitate rigorous safety evaluations to ensure that their delivery does not harm patients or caregivers.^[^ ^134^ ^]^ On an encouraging note, ongoing preclinical studies are steadily building a foundation of knowledge about how tumors respond to redox modulation. This is an inherently interdisciplinary effort: progress will hinge on collaboration among experts in redox biology (to understand the complex responses of cells), materials scientists and chemical engineers (to design better nanocarriers and sensors), and clinical oncologists (to identify the scenarios and tumor types where these approaches could be most beneficial). Several scientific questions remain open, offering rich opportunities for future exploration. For example, to what extent does inducing reductive stress influence anti‐tumor immunity? Could a carefully controlled reductive stress within tumor cells trigger immunogenic cell death that helps rally the immune system against cancer, or will it predominantly cause immunosuppression as seen in some contexts? Also, how does reductive stress impact metastasis in various cancer types? Does forcing disseminated tumor cells into a highly reduced state hinder their survival, or could it inadvertently aid them in establishing new colonies? Addressing such questions will help refine the therapeutic windows.

In conclusion, while challenges remain, the ability to manipulate the redox environment of cancer cells represents a new frontier in cancer therapy. By precisely tilting the redox balance and pushing cancer cells into a state of reductive stress that they cannot tolerate, future treatments could selectively eliminate malignant cells while sparing normal ones. This paradigm turns an underappreciated aspect of cellular physiology into a powerful strategy against cancer. As our understanding deepens and technology advances, nanomedicine‐driven modulation of reductive stress could become a valuable addition to the cancer therapy arsenal, aligning with the broader vision of precision medicine to exploit specific vulnerabilities of a patient's tumor for maximum therapeutic gain.

## Conflict of Interest

The authors declare no conflict of interest.
